# PCBP2-dependent secretion of miRNAs via extracellular vesicles contributes to the EGFR-driven angiogenesis

**DOI:** 10.7150/thno.102391

**Published:** 2025-01-01

**Authors:** Hou-Fu Xia, Xiao-Le Wang, He-Jing Zhang, Kui-Ming Wang, Lin-Zhou Zhang, Yang Yang, Xin Shi, Gang Chen

**Affiliations:** 1 State Key Laboratory of Oral & Maxillofacial Reconstruction and Regeneration, Key Laboratory of Oral Biomedicine Ministry of Education, Hubei Key Laboratory of Stomatology, School & Hospital of Stomatology, Wuhan University, Wuhan 430079, China.; 2 Department of Oral and Maxillofacial Surgery, School and Hospital of Stomatology, Wuhan University, Wuhan 430079, China.; 3 Center of Stomatology, Tongji Hospital, Tongji Medical College, Huazhong University of Science and Technology, Wuhan 430030, China.; 4 TaiKang Center for Life and Medical Sciences, Wuhan University, Wuhan 430071, China.; 5 Frontier Science Center for Immunology and Metabolism, Wuhan University, Wuhan 430071, China.

**Keywords:** epidermal growth factor receptor, extracellular vesicles, angiogenesis, miRNA, sorting, poly(rC) binding protein 2, oral squamous cell carcinoma

## Abstract

**Rationale:** The EGFR-driven angiogenesis is crucial in solid tumors, particularly through the delivery of biomolecules via extracellular vesicles (EVs), but the mechanism by which EGFR regulates EV cargo is still unclear.

**Methods:** First, cell co-culture and murine tumor models were employed to examine the impact of EGFR overexpression on the pro-angiogenic properties of small EVs (sEVs) derived from oral squamous cell carcinoma (OSCC). Small RNA sequencing was then used to compare the miRNA profiles of OSCC-sEVs with and without EGFR overexpression, followed by functional enrichment and motif analyses of the differentially expressed miRNAs. Next, miRNA pull-down assays were conducted to identify potential molecules involved in sorting these miRNAs. Finally, the role of the candidate sorting protein was validated using existing public database, tissue samples, cell lines, and murine tumor models.

**Results:** EGFR overexpression significantly enhances the pro-angiogenic effects of OSCC-sEVs, accompanied by a marked increase in the content of nucleic acid cargo carried in these sEVs. Small-RNA sequencing identified a group of miRNAs that were significantly enriched in OSCC-sEVs due to EGFR overexpression, which primarily functioned in angiogenesis and shared a characteristic “GGGU” motif. EGFR overexpression also strengthened the binding of PCBP2 with miRNAs containing this “GGGU” motif, thereby promoting their secretion through sEVs to support tumor angiogenesis. Mechanismly, EGFR overexpression upregulates PCBP2 protein content by activating its transcription rather than regulating the mRNA stability in OSCC cells. Additionally, depletion of PCBP2 impaired the EGFR-driven tumor angiogenesis by inhibiting the secretion of pro-angiogenic miRNAs through sEVs.

**Conclusions:** EGFR boosts PCBP2 expression via transcriptional regulation, which then promotes the loading of specific miRNAs into sEVs by binding to the “GGGU” motif, thereby driving tumor angiogenesis.

## Introduction

Epidermal growth factor receptor (EGFR) is a receptor tyrosine kinase that is overexpressed in a variety of human solid tumors, especially epithelial malignancies, including carcinomas of the lung 1, colon 2, brain 2, 3, and head and neck 4. Common factors that lead to EGFR overexpression include gene amplification 5, DNA methylation 6, mutation 7, and degradation inhibition 8. Cells that overexpress EGFR are not only more sensitive to the activation of growth factors, but also maintain a continuous autoactivation that is not dependent on ligands. There is ample documentary evidence of EGFR overexpression in triggering malignant transformation, as well as numerous studies on the tumorigenicity of cell lines expressing activated EGFR in mouse models 9. The observation of heightened EGFR expression in cancer cells, coupled with the established correlation between EGFR overexpression and reduced patient survival, has spurred the development of numerous therapeutic strategies aimed at specifically targeting EGFR. However, the results of existing clinical trials to date suggest that targeting EGFR alone may not be sufficient to eradicate established tumors 10, 11. Further mechanistic studies suggest that EGFR overexpression is not an isolated event. Beyond its role in regulating the phenotype and function of malignant cells, EGFR also plays a crucial role in modifying the external microenvironment to drive the development of tumors, particularly by promoting tumor angiogenesis 12.

Tumor angiogenesis refers to the process by which new blood vessels are formed from existing vasculature. It is an early and pivotal response during tumor initiation, essential for the sustained growth and progression of neoplasms 13. The formation of new blood vessels not only provides the tumor with a continuous supply of oxygen and nutrients, but also facilitates the removal of metabolic waste products, enabling rapid tumor expansion. Moreover, the newly formed vasculature serves as a conduit for tumor cells to disseminate to distant sites through metastasis. Therefore, the vascular system plays a critical role in tumor development by supporting the dynamic metabolism and growth demands of cancer cells 13, 14. Tumor cells exert significant regulatory influence on angiogenesis primarily through paracrine signaling pathways. Cytokines (VEGF, IL-8) are key mediators released by tumor cells to stimulate the vessel formation in the tumor microenvironment. A cycle of positive feedback occurs, where EGFR signaling boosts VEGF expression, and VEGF, in turn, amplifies EGFR activity, thereby strengthening this interaction 15. miRNAs and MMPs also are key regulators of tumor angiogenesis. The former regulates the pro-angiogenic signaling network from the protein expression level inside the target cells 16, while the latter guides the path of vessel sprouting through remodeling of extracellular matrix 16, 17. Recently, extracellular vesicles (EVs) have become pivotal in clarifying tumor angiogenesis due to their remarkable stability, diversified cargos 18, and precise targeting 19.

EVs are small membrane-bound particles released by cells into the extracellular environment 20. They play crucial roles in regulating neighboring or distant recipient cells in various physiological and pathological processes by delivering a diverse array of biomolecules, including proteins, lipids, and nucleic acids (notably microRNAs). EVs-mediated cellular communication has become a highly discussed area in cancer research recently. EVs can be regarded as trillions of miniature replicas of tumor cells, which selectively inherited the traits of their parent cells and specifically amplified the disease-promoting potential of tumor cells throughout the body 20, 21. The characteristics stem from the cargo profile possessed by EVs, which is likely intentionally designed by parental tumor cells. On one hand, the biomolecules carried by EVs serve as crucial markers for identifying the origin of their parent cells. While there may be slight variations in the molecular traits between EVs and their parent cells, EVs often retain distinct molecules that provide opportunities for sensitive diagnosis and continuous monitoring of specific tumors. For example, Glypican-1 (GPC-1) is utilized to detect cancer EVs and is employed for early detection of pancreatic cancer 22. On the other hand, the abnormal accumulation of specific biomolecules in EVs explains a fundamental mechanism that governs tumor progression or contributes to resistance to treatments. The release of PD-L1 by tumor cells through EVs has been recognized as a major mechanism for inducing systemic immune suppression and diminishing the immunotherapy efficacy in a variety of tumors 23. This highlights the importance of revealing the characteristic cargo profile and biogenesis mechanism of EVs for tumor pathophysiology and clinical transformation.

Due to variations in the composition and quantity of cargos (proteins, nucleic acids) identified in EVs compared to their parent cells, existing studies tend to suggest that biomolecules are likely deliberately and selectively packaged into EVs during the biogenesis process 24, 25. The sorting mechanisms of protein cargos were well-developed, and which could be broadly categorized into the endosomal sorting complex required for transport (ESCRT)-dependent or non-ESCRT-dependent pathway 26. Cargo sorting occurs at the initial phase of EV biogenesis. The key relies on the targeted recognition of the protein cargo by a specialized sorting protein, followed by the incorporation of this newly formed complex into endosomes. Known regulators responsible for protein cargo sorting include hepatocyte growth factor-regulated tyrosine kinase substrate (HRS) 27, Alix, RAB31 28, etc. However, despite the extensive interest in nucleic acid cargos in both fundamental research and clinical applications 29, the sorting mechanism for nucleic acid cargos remains less understood 29, 30. Inspired by the insights from protein cargos, a few studies have attempted to identify sorting proteins specific to certain EV miRNAs recently 31, 32. These studies provide a framework for understanding how EGFR signaling promotes tumor progression by regulating EV-mediated intercellular communication 33. The EGFR signaling has been revealed to regulate miRNA biogenesis 34, 35, while previous studies, including ours, have demonstrated that EV miRNAs promote critical tumorigenic processes. However, the precise mechanisms through which EGFR determines the selective sorting of oncogenic miRNAs to exert a pro-tumorigenic effect remain unclear.

Here, we comprehensively compared the impact of EGFR overexpression on miRNA profile in both tumor cells and their derived EVs. Our findings confirmed the EGFR-driven secretion of angiogenesis-associated miRNAs and identified short sequence motifs (EVmotifs) in the miRNAs that guide their loading into EVs. Additionally, we also screened for sorting proteins responsible for the selective packaging of these angiogenesis-related miRNAs. We confirmed that PCBP2 plays a crucial role in promoting EGFR-driven tumor angiogenesis both *in vivo* and *in vitro*, by controlling the secretion of these EVmotif-containing miRNAs. In summary, we have unveiled the pivotal role and underlying mechanism of EGFR in promoting tumor angiogenesis by controlling the PCBP2-dependent miRNA sorting into sEVs. This study provides a theoretical foundation for enhancing our understanding of the pathophysiology in epithelial malignancies and offers a potential target for developing EV miRNA-based therapeutic strategies.

## Methods

### Clinical Samples

The study was conducted in accordance with the Declaration of Helsinki and approved by the Medical Ethics Committee of the Hospital of Stomatology, Wuhan University. Tissue samples of 175 OSCC patients (**Table S1**) and 25 healthy donors were used for the tissue microarray (TMA) analysis. Fresh tumorous tissues and normal oral mucosa of the same OSCC patient were also obtained for the isolation of Ti-sEVs. All patients provided informed consent.

### Cell culture

HEK293T and CAL27 cells were cultured in high glucose Dulbecco's modified eagle medium (DMEM, Gibco) containing 10% fetal bovine serum (FBS, PAN) and regularly tested for mycoplasma contamination. HEK293T cells were cultured to produce lentivirus for the stable construction of cell lines. The CRISPR-Cas9 system was adopted to deplete PCBP2 in CAL27 cells (sgRNA sequences: GCTTTGAAGATGGCATTAGT and TTTGTCAATGATCATAGCAA).

### Immunohistochemistry (IHC)

The paraffin-embedded tissue samples were sectioned at 4 μm. After deparaffinization and rehydration, the sections were subjected to heat-induced epitope retrieval. Samples were blocked with goat serum and then incubated with indicated primary antibodies at 4 °C overnight. After washing three times with phosphate buffered saline (PBS), tissue sections underwent incubation with a horseradish peroxidase-conjugated secondary antibody (Mxb Biotechnologies). Finally, the staining visualization were performed with DAB (Mxb Biotechnologies) and hematoxylin (Servicebio) counterstaining.

### Immunocytofluorescence

CAL27 cells were seeded in 8 mm coverslips and fixed, followed by permeabilization in 0.2% Triton X-100 (Biosharp) for 15 min at room temperature (RT). The coverslips were blocked with 3% bovine serum albumin (BSA, BioFroxx) for 1 h and then incubated with indicated primary antibodies overnight at 4 °C. After washing 3 times with PBS, the coverslips were further incubated with diluted secondary antibodies for 1 h at RT. Nuclei were stained with Hoechst 33342 (ThermoFisher). The coverslips were mounted with antifade mountant (Beyotime) and visualized with a spinning-disk confocal microscope system (AndorRevolution) at 100 × magnification. The “co-localization” module of the ImageJ program was performed for co-localization analysis.

### Fluorescence *in situ* hybridization (FISH)

The expression of miR-3189-3p in human tissues was detected using the hsa-miR-3189-3p Detection Kit (Boster Bio-Engineering Company, Wuhan, China) according to the manufacturer's protocol. The positive signal of hsa-miR-3189-3p expression was visualized with a spinning-disk confocal microscope system (AndorRevolution).

### Isolation and characterization of sEVs

For isolation of CAL27 cells-derived sEVs, the conditioned media was collected and centrifuged at 400 g for 10 min, 2000 g for 15 min, and 15,000 g for 30 min at 4 °C. Finally, the supernatant underwent ultracentrifugation at 120,000 g for 75 min at 4 °C (Optima XE-100, Beckman), and the precipitate was resuspended in sterile PBS, followed by another ultracentrifugation (120,000 g, 75 min, 4 °C) to collect the sEVs. The obtained sEVs were stored at -80 °C for use.

For Ti-sEVs, the samples were minced into pieces and treated with dissociation solution (1 mg/ml collagenase IV and 0.1 mg/ml DNases I) at 37 °C for 1 h. The dissociation solution was then filtered through a 70 μm strainer, followed by centrifugation at 300 g, 2000 g, and 10,000 g at 4 °C, and finally centrifuged at 120,000 g for 70 min (Optima XE-100, Beckman). The pellets (sEVs) were resuspended and stored at -80 °C.

For nanoparticle tracking analysis (NTA), the sEVs were diluted with PBS and tested for the size distribution and concentration on the ZetaView (Particle Metrix). For transmission electron microscopy (TME), the sEVs solution were dropped onto formvar carbon coated nickel grids and stained with 2% uranyl acetate. After air-dried, the grids were visualized using a JEM-1011 transmission electron microscope (Hitachi).

### Western blotting

Cells and sEVs were lysed with a lysis buffer (20 mM Tris-HCl-pH 7.5, 150 mM NaCl, 1 mM Na2EDTA, 1 mM EGTA, 1% Triton, 2.5 mM sodium pyrophosphate, 1 mM β-glycerophosphate, 1 mM Na_3_VO4, and 1 µg/mL leupeptin) supplemented with freshly added inhibitors for protease and phosphatase (MedChem Express). Protein concentration was calculated using a BCA assay kit (Beyotime). Samples were separated in 10% SDS-PAGE gel and transferred onto a PVDF membrane (Roche). Proteins were immunoblotted with indicated antibodies and detected with ECL system.

### miRNA pull-down assay

Cells were lysed with RIPA buffer (Beyotime) containing inhibitors for protease, phosphatase and RNase, and supplemented with biotin-labeled miRNA mimics (Sangon Biotech). The mixture was incubated on a rotator at 4 °C for 4 h and followed by the adding of streptavidin-agarose beads (Sigma). After incubation for 4 h and washing for 6 times, the beads were obtained for electrophoresis or proteomic analysis.

### Silver staining

Protein samples were mixed with 5× SDS-PAGE loading buffer (Beyotime) before the electrophoresis. After electrophoresis, the gel was immersed in a fix solution (50 mL ethanol, 10 mL acetic acid and 40 mL Milli-Q grade pure water), and incubated at room temperature with a shaking speed of 70 rpm. Then, the experiments were conducted according to the instructions of a silver stain kit (Sangon).

### Mass Spectrometry

The precipitations were lysed and mixed with TEAB buffer. To each sample, Lys-C enzyme was added in a ratio of 100:1, and incubated at 37 °C for 3 h.Trypsin was added in a ratio of 50:1, and incubated at 37 °C for 16 h. The zymolysis was terminated by adding 10 % TFA. Ethyl acetate was added in five volumes to each sample. The samples were then centrifuged, freeze-dried, and desalted. An appropriate amount of peptide from each sample was subjected to chromatography using an Easy-nLC 1200 chromatography system (Thermo Fisher Scientific). DIA mass spectrometry was performed on a Q-Exactive HF-X mass spectrometer following peptide separation.

### Immunoprecipitation (IP)

Cells were lysed with an IP lysis buffer (Beyotime) containing inhibitors for protease, phosphatase and RNase. 1 mg proteins of each sample were incubated with the Flag-conjugated agarose beads (Sigma) followed by rotation at 4 °C for 6 h. After washing 5 times with IP lysis buffer on rotator, precipitations were treated with TRIzol (Takara) for the isolation of RNA.

### RNA isolation and real-time quantitative polymerase chain reaction (RT-qPCR)

Equal particles of sEVs were used in each group, and the total RNA was extracted using TRIzol Reagent (Takara) according to the manufacturer's instructions. The total RNAs were quantified with a Nanodrop (ThermoFisher) before the analysis with PCR or Agilent 2100 Bioanalyzer. For miRNA detection, 250 ng RNA was used for the 1st Strand cDNA synthesis and real-time PCR was performed by using miRNA Universal SYBR qPCR Master Mix (Vazyme). All the primers were purchased from GeneCopoeia. The quantitative analysis was performed on a LightCycler® 480 Instrument (Roche) according to standard conditions. Primer sequences shown in **Table 1**.

### mRNA Stability assay

Cells exposed to the transcription inhibitor actinomycin D (2 µg/mL, Selleck, USA) to block the synthesis of new mRNA were harvested at various time points. mRNA levels for each sample were normalized to the levels measured at the starting point.

### Electroporation

The loading of miRNA mimics or RNase A into sEVs were realized using the Bio-Rad Gene Pulser Xcell Electroporation System. sEVs and indicated agents were mixed with electroporation buffer (Bio-Rad) in 0.4 cm cuvettes (Bio-Rad). Electroporations were performed with parameter setting at 250 V and 350 μF. Then, the mixture was incubated at 37 °C for 20 min and centrifuged at 120,000 g for 70 min (Optima XE-100, Beckman) in PBS to remove the excess free agents.

### Tube formation assay

HUVECs were pretreated with indicated conditions for 24 h and separately seeded into 24-well plate (8 × 10^4^ cells per well) which were precoated with Matrigel (BD Biosciences). The HUVECs were cultured under normal conditions and the capillary-like structures were recorded under a phase microscope (Nikon). The branching points and total tube length were quantified by angiogenesis analyzer plugin of ImageJ.

### Flow cytometry

CAL27 cells were loaded with FAM-labeled miRNA mimics (Sangon) through Lipo3000 (ThermoFisher) and followed by the isolation of sEVs from the supernatant. The transfection efficiency was measured with a CytoFLEX Flow Cytometer (Beckman). The isolated sEVs were analyzed on an Apogee A-50 Micro Flow Cytometer (Apogee Flow Systems) at a flow rate of 1.5 μL min^-1^.

### *In vivo* assay

All animal experiments were approved by the Institutional Animal Care and Use Committee (IACUC) of Hospital of Stomatology Wuhan University. 7-week-old nude mice were purchased from Slack Jingda Laboratory Animal Co., Ltd (Hunan, China) and housed at the Specific Pathogen Free Animal Experiment Center of Hospital of Stomatology Wuhan University. 2 × 10^6^ CAL27 cells (Parental or PCBP2 KO) were injected subcutaneously into the dorsal part of mice. Tail vein injections of sEVs (100 μg in 150 μl PBS) were performed every 3 days. Tumors were measured using a digital caliper and the volume was calculated using the following formula: (length × width × width)/2. Mice were euthanized at the indicated timepoint or if the longest dimension of the tumors reached 2.0 cm.

### Statistical analysis

Statistical analysis was performed with GraphPad Prism software. All results were expressed as mean ± standard deviation. Two-tailed paired or unpaired student's t-test was used to compare the difference between two groups and one-way analysis of variance (ANOVA) followed by Tukey's multiple comparison test were performed to compare the differences between multiple groups, or as indicated in the figure legend.

## Results

### RNA cargo within sEVs is crucial for EGFR-driven tumor angiogenesis

**Figure 1:**
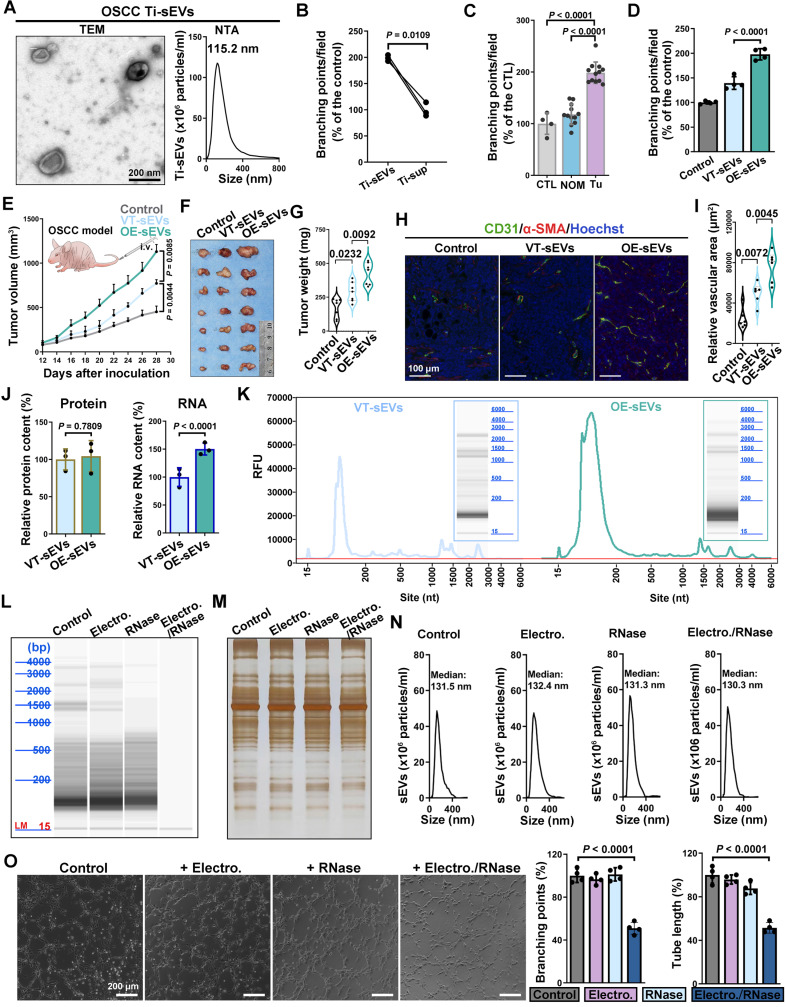
** RNA cargo within sEVs is crucial for EGFR-driven tumor angiogenesis. (A)** The representative TEM image and NTA result of sEVs isolated from OSCC tissues. Scale bar, 200 nm. **(B)** Comparing the pro-angiogenic ability of Ti-sEVs with paired leach supernatant obtained from the same OSCC tissues through the tube formation assay. **(C)** Comparing the pro-angiogenic ability of Ti-sEVs obtained from tumor tissues (Tu) and paired normal mucosa (NOM) through the tube formation assay. **(D)** Comparing the pro-angiogenic ability of sEVs derived from control (VT-sEVs) and EGFR-overexpressed (OE-sEVs) CAL27 cells through the tube formation assay. **(E)** The tumor growth of CAL27 xenograft tumors in BALB/c nude mice after injections of sEVs from control (VT-sEVs) and EGFR-overexpressed (OE-sEVs) CAL27 cells. The CAL27 xenograft tumors were harvested **(F)** and weighed **(G)** at the indicated timepoint. **(H)** The representative image of CD31^+^α-SMA^+^ vessels in the tumor tissues. Scale bar, 100 μm. **(I)** Quantitative analysis of microvessel density in the xenograft tumors. **(J)** Quantitative analysis of mean protein and RNA content in sEVs derived from control (VT-sEVs) and EGFR-overexpressed (OE-sEVs) CAL27 cells. **(K)** Representative electrophoresis results of total RNA isolated from sEVs. Representative electrophoresis **(L)** and silver stain **(M)** results of total RNA isolated from sEVs which were treated with the indicated agents. **(N)** NTA results of the sEVs after treatment with the indicated agents. **(O)** Comparing the pro-angiogenic ability of sEVs after treatment with the indicated agents through the tube formation assay. Scale bar, 200 μm.

Oral squamous cell carcinoma (OSCC) is a typical epithelial malignancy characterized by active tumor angiogenesis 36. To compare the pro-angiogenic abilities, both EVs and leach supernatant were obtained from fresh OSCC tissues. The EVs isolated from OSCC tissue (Ti-EVs) exhibited typical saucer-shaped morphology with a diameter less than 200 nm (defined as sEVs according to recommendations of the International Extracellular Vesicle Association) (Figure 1A). Compared with the leach supernatant, OSCC Ti-sEVs obtained from equal tumor tissues displayed a more significant pro-angiogenic effect (Figure 1B). Furthermore, compared to sEVs obtained from paired normal mucosa, tumor-derived Ti-sEVs exhibited distinctive pro-angiogenic properties (Figure S1A and Figure 1C), which were positively associated with the abundance of EGFR expression in their parent tumor cells (Figure S1B-C). This finding was supported by the positive correlation between EGFR content and microvessel density in OSCC (Figure S2A-C). Elevated EGFR expression is another typical feature of OSCC (Figure S2A). We then isolated sEVs from culture supernatant of CAL27 cells to verify the promotion of EGFR-overexpression on the pro-angiogenic effects of OSCC-derived sEVs *in vitro* and *in vivo* (Figure S3A-C). sEVs-derived from EGFR-overexpressed CAL27 cells (CAL27^Hi-EGFR^) not only significantly promoted the formation of tube-like structures of vascular endothelial cells (Figure S3D and Figure 1D), but also significantly increased the intra-tumor microvessel density in murine OSCC models, resulting in more rapid tumor growth (Figure 1E-I). The same results were found in 2 different OSCC cell lines (Figure S3E-F).

Next, we tried to identify the factors by which EGFR-overexpressed tumor cells enhance the pro-angiogenic effects of their sEVs. We initially compared the alterations in the primary contents carried by sEVs following the overexpression of EGFR in their originating cells. The results showed that sEVs derived from CAL27^Hi-EGFR^ cells carried a more abundant RNA cargo, but the total protein content did not change significantly (Figure 1J). Nucleic acid electrophoresis results further revealed that EGFR overexpression mainly promoted the loading of small RNA (sRNA, <200 bp) into sEVs (Figure 1K). The phospholipid membrane provides excellent protection for the cargos inside the sEVs. To explore the role of the increased sRNA cargo in the pro-angiogenic effect, the electroporation strategy was applied to assist the infiltration of RNase into sEVs. The combined treatment of electroporation and RNase almost completely destroyed the sRNA cargo carried by CAL27^Hi-EGFR^ cells-derived sEVs (Figure 1L), but did not significantly affect their protein content (Figure 1M) and size distribution (Figure 1N). Specific depletion of RNA in OSCC sEVs significantly impaired their pro-angiogenesis ability (Figure 1O), indicating that enhanced loading of sRNA may be the key mechanism by which tumor cells with elevated EGFR expression promote angiogenesis through sEVs.

### EGFR promotes the loading of angiogenesis-associated miRNAs into sEVs

**Figure 2:**
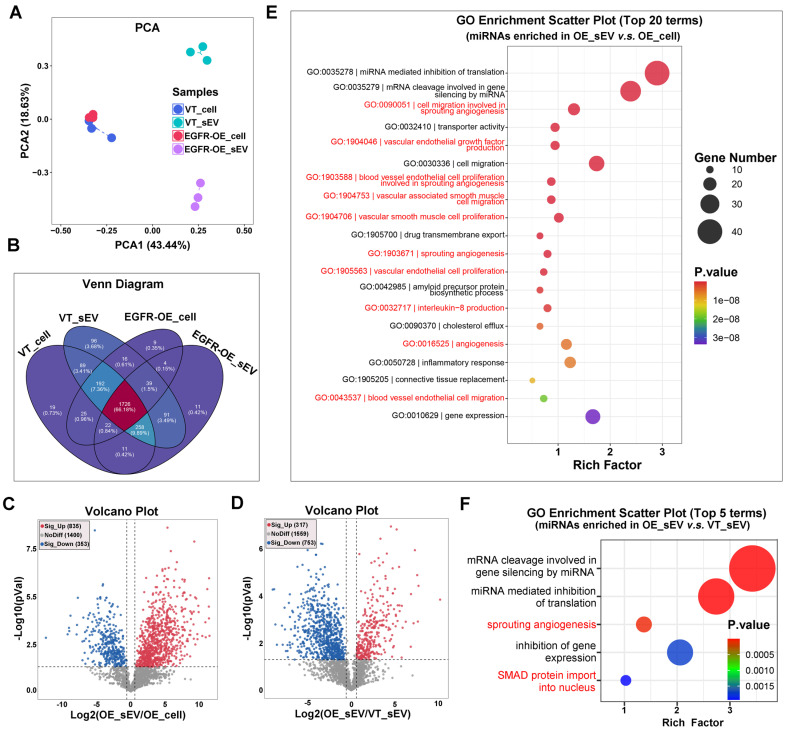
** EGFR promotes the loading of angiogenesis-associated miRNAs into sEVs. (A)** Principal Component Analysis (PCA) of miRNAs isolated from control (VT) and EGFR-overexpressed (EGFR-OE) CAL27 cells and their derived sEVs. **(B)** Venn Diagram displayed the overlapping sets of identified miRNAs in control (VT) and EGFR-overexpressed (EGFR-OE) CAL27 cells and their derived sEVs. **(C)** Volcano Plot showed the difference in miRNA abundance in EGFR-overexpressed (EGFR-OE) CAL27 cells (OE_cell) and the derived sEVs (OE_sEV). **(D)** Volcano Plot showed the difference in miRNA abundance in sEVs isolated from control (VT_sEV) and EGFR-overexpressed (VT_sEV) CAL27 cells. The GO enrichment analysis of miRNAs enriched in sEVs isolated from EGFR-overexpressed CAL27 cells (OE_sEV) when compared with their parental cells (OE_cell) **(E)** or sEVs isolated from control CAL27 cells (VT_sEV) **(F)**.

Recognizing the critical significance of sEV sRNA in EGFR-driven angiogenesis, we employed sRNA Sequencing to comprehensively examine how EGFR overexpression in OSCC cells affects the enrichment of sRNAs into sEVs. The alignment results of sRNA Sequencing data revealed that miRNA was the main type of sRNA carried by CAL27 sEVs. EGFR overexpression exerts a more striking impact on miRNA content in sEVs compared to the parental cells (Figure 2A). Specifically, with the overexpression of EGFR in CAL27 cells, 48 new types of miRNA were identified in the sEVs, and the type of miRNAs that were almost completely sorted into sEVs increased from 87 to 371 (Figure 2B). Not only miRNA types, but also the miRNA content in sEVs were affected by EGFR overexpression in OSCC cells: the absolute abundance of total 1188 miRNAs in EVs were inconsistent with that in the parental cells (835 up-regulated and 353 down-regulated) (Figure 2C); moreover, the enrichment of an additional 317 miRNAs within the sEVs were notably enhanced by EGFR overexpressing in the parental cells (Figure 2D). These findings indicate that EGFR overexpression promotes the active sorting of specific miRNAs into sEVs in OSCC cells. Functional analysis of differential miRNAs between sEVs and their parental cells revealed that sEVs-mediated miRNA secretion in OSCC cells predominantly participate in angiogenesis process (Figure 2E). These miRNAs mainly regulate endothelial cells, perivascular cells, extracellular matrix, cytokines, and other related components. Functional analysis of differential miRNAs in sEVs derived from CAL27 cells with varying EGFR levels further substantiated the role of EGFR-induced miRNA secretion in enhancing tumor angiogenesis (Figure 2F).

### Specific sequence of miRNAs dictates their EGFR-driven loading into sEVs

**Figure 3:**
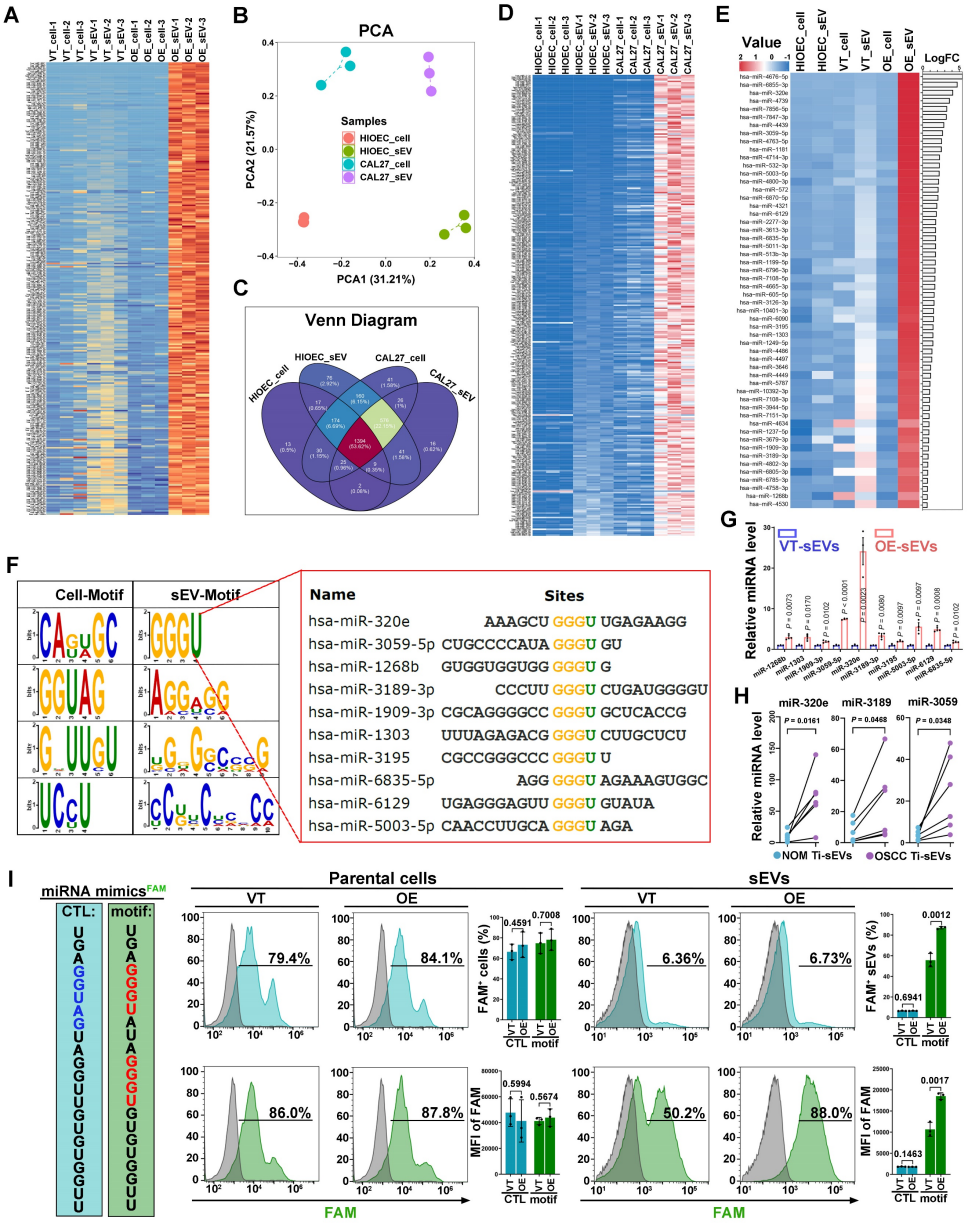
** Specific sequence of miRNAs dictates their EGFR-driven loading into sEVs. (A)** The heat map displayed the expression pattern of screened 280 miRNAs in control (VT_cell) and EGFR-overexpressed CAL27 cells (OE_cell) and the derived sEVs (VT_sEV, OE_sEV). **(B)** PCA of miRNAs isolated from normal (HIOEC) and malignant oral epithelial cells (CAL27) and their derived sEVs. **(C)** Venn Diagram displayed the overlapping sets of identified miRNAs in normal (HIOEC) and malignant oral epithelial cells (CAL27) and their derived sEVs. **(D)** The heat map displayed the expression pattern of screened 315 miRNAs in normal (HIOEC) and malignant oral epithelial cells (CAL27) and their derived sEVs. **(E)** The heat map displayed the expression level of candidate 54 miRNAs in sEVs isolated from EGFR-overexpressed CAL27 cells. **(F)** Over-represented sequences identified in those 54 candidate miRNAs (sEV-Motif). **(G)** The enrichment of 10 GGGU motif-containing miRNAs in sEVs isolated from EGFR-overexpressed CAL27 cells were verified by PCR. **(H)** Comparing the levels of miR-320e, miR-3189 and miR-3059 in Ti-sEVs obtained from tumorous tissues and paired normal oral mucosa (NOM) of OSCC patients, n = 6. **(I)** Verifying the effects of sEV-Motif on the secretion of miRNAs by transfecting synthetic GGGU motif-containing miRNA analogues into CAL27 cells.

Several studies have proposed that selective enrichment of biomolecules in sEVs results from interactions between cargo and specific regulators 32. We then aimed to investigate whether a similar mechanism exists in the EGFR-driven secretion of angiogenesis-associated miRNAs. Firstly, total 280 types of miRNAs which were significantly enriched in CAL27 cells-derived sEVs resulting from EGFR overexpression were identified (Figure 3A). To eliminate the interference from lentivirus infection process, we also compared normal oral mucosal epithelial cells (HIOECs) with CAL27 cells, which owns the naturally occurring EGFR overexpression during the malignant transformation of OSCC. The malignant transformation of oral mucosal epithelium is also accompanied by changes in miRNA cargo carried by their sEVs (Figure 3B-C). We screened 315 types of miRNAs whose enrichment in sEVs were enhanced by malignant transformation, through comparison of malignant and normal oral mucosal epithelial cells (Figure 3D). Ultimately, we identified 54 types of miRNAs through a cross-comparison of aforementioned 280 miRNAs and 315 miRNAs (Figure 3E). These 54 miRNAs represent types whose sorting into sEVs were enhanced by the upregulation of EGFR expression during the malignant transformation of oral mucosal epithelial cells (the initiation stage of OSCC). These 54 miRNAs share some sequence fragments in common and are distinct from the miRNAs predominantly found in the parental cells. The most notable sequence is the “GGGU” motif (referred as the EGFR-driven sEVmotif), which was shared by 10 types of miRNAs (Figure 3F). PCR results confirmed that EGFR overexpression significantly promoted the secretion of the 10 “GGGU motif-containing” miRNAs via sEVs in different OSCC cells (Figure 3G and Figure S4). High levels of the GGGU motif-containing miRNAs (miR-320e, miR-3189 and miR-3059) were also detected in Ti-sEVs of OSCC patients (Figure 3H). We then transfected artificial GGGU motif-containing miRNA mimics into CAL27 cells and detected the levels of these target miRNAs in secreted sEVs using highly sensitive flow cytometry (Apogee A60) (Figure 3I). The findings confirmed that the GGGU motif is crucial for the EGFR-driven loading of miRNAs into sEVs in OSCC cells.

### Screening regulators responsible for the EGFR-driven secretion of miRNAs

**Figure 4:**
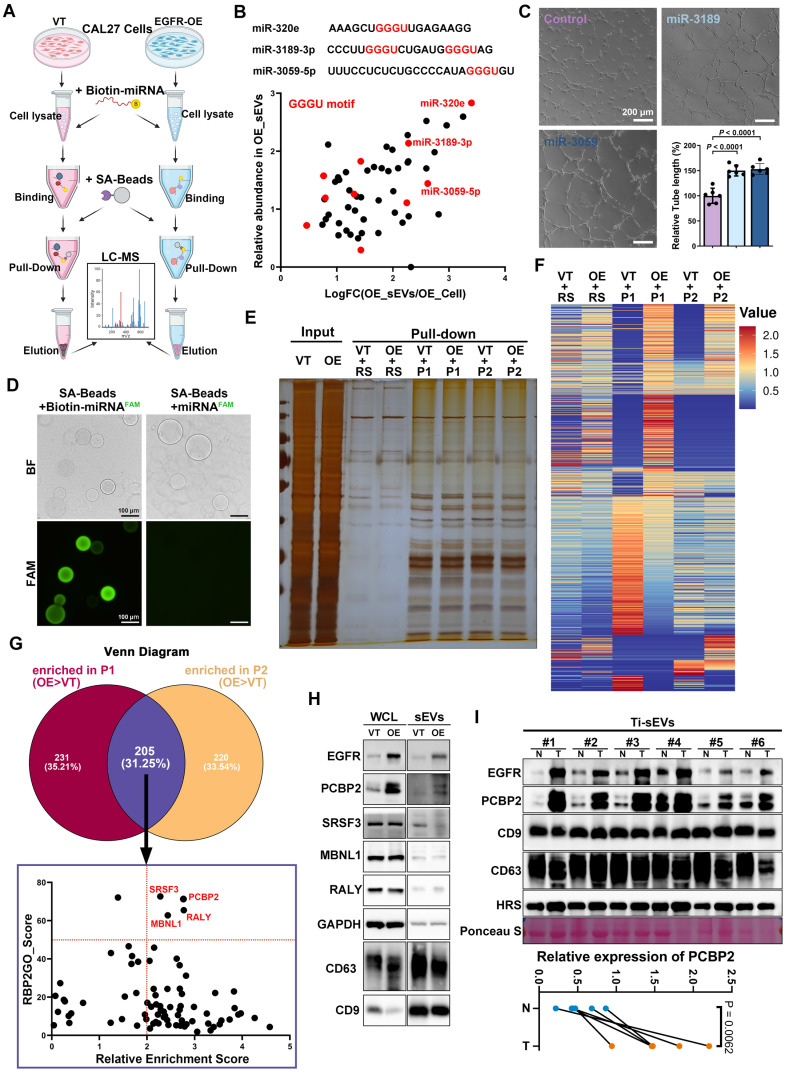
** Screening regulators responsible for the sorting of miRNAs into EGFR^+^ sEVs. (A)** Schematic diagram of the biotin-miRNA pull-down assay. **(B)** Screening the bait miRNAs based on fold change and relative abundance. **(C)** Comparing the pro-angiogenic ability of sEVs with (Electro.) or without (Control) exogenous loading of candidate miRNA mimics through the tube formation assay. Scale bar, 200 μm. **(D)** Representative images of effective enrichment of biotin-miRNAs by SA-beads. Scale bar, 100 μm. **(E)** Representative silver stain image of precipitations pulled by a random sequence (RS), miR-3189-3p (P1) and miR-3059-5p (P2) from control (VT) and EGFR-overexpressing (OE) CAL27 cells. **(F)** The heat map shows the abundance of the identified proteins in precipitations pulled by a random sequence (RS), miR-3189-3p (P1) and miR-3059-5p (P2) from control (VT) and EGFR-overexpressing (OE) CAL27 cells. **(G)** Screening the candidate proteins responsible for EGFR-driven sorting from the overlapping precipitations of miR-3189-3p (P1) and miR-3059-5p (P2). **(H)** The expression levels of candidate sorting proteins were detected by western blotting in control (VT) and EGFR-overexpressed (OE) CAL27 cells (WCL) and their derived sEVs. **(I)** The expression levels of PCBP2 were detected by western blotting in sEVs isolated from tumor tissues (T) and paired normal mucosa (N) of OSCC patients.

Building upon our discovery of the specific sequences involved in EGFR-driven miRNA sorting, we next employed a miRNA pull-down strategy to screen sorting proteins that directly interact with the target miRNAs (Figure 4A). Based on their absolute abundance and functional relevance, we selected miR-3189-3p and miR-3059-5p from the 10 GGGU motif-containing miRNAs (Figure 4B and Figure S5). Both miR-3189-3p and miR-3059-5p were closely associated with angiogenesis regulation (Figure S5). Functional experiments confirmed that loading miR-3189-3p or miR-3059-5p into CAL27 sEVs via electroporation significantly enhanced the tube formation ability of human umbilical vein endothelial cells (HUVECs) (Figure 4C). Biotin-labeled miR-3189-3p (P1) and miR-3059-5p (P2) mimics were synthesized and utilized as bait probes to directly enrich interacting proteins via their specific binding to streptavidin-coated beads (SA-beads) (Figure 4D). The precipitate pulled both in control and EGFR-overexpression CAL27 cells by random sequence (RS), miR-3189-3p (P1) and miR-3059-5p (P2) were subjected to protein mass spectrometry (Figure 4E-F). We individually analyzed proteins whose interaction with miR-3189-3p or miR-3059-5p is enhanced by EGFR overexpression, and then focused on the overlapping ones (Figure 4G). EGFR overexpression significantly increased the abundance of poly(rC) binding protein 2 (PCBP2) both in CAL27 cells and the derived sEVs (Figure 4H). Analysis of Ti-sEVs obtained from OSCC patients further validated that PCBP2 was more abundant in tumor Ti-sEVs compared to paired normal mucosa Ti-sEVs (Figure 4I). These findings suggest that PCBP2 may act as a sorting protein directly involved in EGFR-driven secretion of pro-angiogenic miRNAs via sEVs in OSCC.

### PCBP2 controls the sorting of miRNAs into sEVs

**Figure 5:**
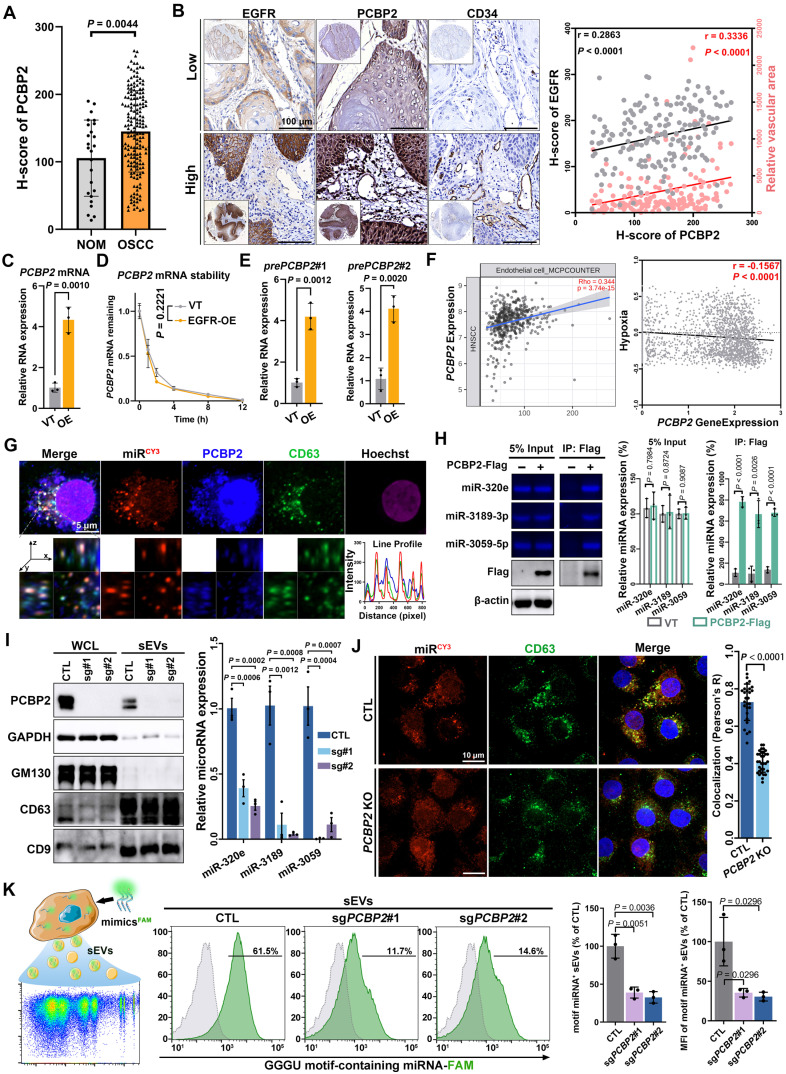
** PCBP2 controls the sorting of miRNAs into sEVs. (A)** The expression level of PCBP2 in normal oral mucosa (NOM) and OSCC tissues were detected by immunohistochemistry. **(B)** Representative images of IHC staining of EGFR, PCBP2 and CD34 in OSCC tissues. Scale bar, 100 μm. **(C)** The mature mRNA level of *PCBP2* in EGFR-overexpressed CAL27 cells was detected by RT-qPCR. **(D)** RT-qPCR was performed to assess the stability of mature mRNA of *PCBP2* in CAL27 cells with (EGFR-OE) or without (VT) EGFR-overexpression. **(E)** The precursor mRNA level of *PCBP2* in CAL27 cells with (EGFR-OE) or without (VT) EGFR-overexpression were detected by RT-qPCR. **(F)** Analyzing the correlation between *PCBP2* expression and endothelial cell infiltration or hypoxia with a multi-omic Atlas of Oral Carcinogenesis database (TIMER 2.0). **(G)** Representative images of immunocytofluorescence staining of CY3-labeled miR-3189, PCBP2 and CD63 in CAL27 cells, the lower panel is a section view of the Z-axis scan. Scale bar, 5 μm. **(H)** Quantifying the content of miR-320e, miR-3189 and miR-3059 in the precipitate pulled by PCBP2-Flag from CAL27 cells with gel electrophoresis. **(I)** The effects of PCBP2 knockout in CAL27 cells on the secretion of miR-320e, miR-3189 and miR-3059 via sEVs were detected by RT-qPCR. **(J)** Representative images of immunocytofluorescence staining of CY3-labeled miR-3189 and CD63 in CAL27 cells with (*PCBP2* KO) or without (CTL) PCBP2 depletion. Scale bar, 10 μm. **(K)** The effects of PCBP2 knockout on the secretion of GGGU motif-containing miRNA was studied by highly sensitive flow cytometry.

The protein level of PCBP2 was significantly upregulated (Figure 5A) and positively correlated with both EGFR content and microvessel density (Figure 5B) in OSCC. PCBP2, a nucleic acid-binding protein, displayed elevated mRNA levels in a variety of tumors (Figure S6A). Moreover, the mRNA levels of *EGFR* and* PCBP2* showed a positive correlation across different types of tumors (Figure S6B), including head and neck squamous cell carcinoma (HNSCC) (Figure S6C). The PCR results also confirmed the elevated mature mRNA level of *PCBP2* in CAL27 cell after EGFR overexpression (Figure 5C). The EGFR-driven PCBP2-upregulation was also found in 2 different OSCC cell lines (SCC25 and HSC-3) (Figure S6D). To assess if EGFR regulates PCBP2 transcription, we interrogated the precursor mRNA (pre-mRNA) expression as opposed to PCBP2 mature mRNA (Figure S6E). Pre-mRNA, also called primary transcript, is the first form of RNA synthesized in the nucleus before splicing, thus more faithfully representing the transcription rate 37. We observed that EGFR overexpression notably enhanced the transcription of *PCBP2* in various OSCC cell lines, without affecting mRNA degradation, as shown by the increased levels of pre-mRNA levels (Figure 5D and Figure S6F-G) and the unchanged half-life of mature mRNA (Figure 5E and Figure S6F-G). Suggesting that EGFR-overexpression transcriptionally regulates PCBP2 expression in OSCC. Another multi-omic Atlas of Oral cancer data (CancerSEA) further validated the increased transcripts of *PCBP2* in malignant oral epithelial cells, which was observed consistently in tumor cells across all 15 oral cancer patients examined (Figure S7A-B). Elevated *PCBP2* levels were positively correlated with higher endothelial cell infiltration and improved hypoxia (Figure 5F), which also was verified in multiple solid tumors (Figure S7C). These results suggested the important role of PCBP2 in EGFR-driven tumor angiogenesis.

The results of fluorescence confocal microscopy clearly displayed strong co-localization of PCBP2, miR-3189 and CD63 in the cytoplasm of CAL27 cells (Figure 5G). Co-immunoprecipitation further confirmed the interaction between PCBP2 and GGGU motif-containing miRNAs (Figure 5H). The FISH experiments in clinical OSCC tissues also demonstrated the regulatory effects of PCBP2 on miRNA loading (Figure S8). Knockout of PCBP2 significantly reduced the levels of GGGU motif-containing miRNAs in CAL27 sEVs (Figure 5I). The immunocytofluorescence analysis confirmed that PCBP2 depletion disrupted the co-localization between miRNAs and CD63 (Figure 5J). The results of highly sensitive flow cytometry provided additional confirmation that PCBP2 depletion hindered the packaging of GGGU motif-containing miRNAs into sEVs. Depleting PCBP2 in EGFR-overexpressed OSCC cells significantly reduced the proportion of GGGU motif-containing miRNA^+^ sEV subgroups and the average amount of GGGU motif-containing miRNA per sEV particle (Figure 5K and Figure S9A-C). Similar results were observed with miR-3189-3p mimics (Figure S9D). In conclusion, EGFR overexpression potentially stimulates the expression of PCBP2 through transcriptional regulation, and PCBP2 subsequently identifies GGGU motif-containing miRNAs to facilitate their secretion via sEVs, thereby promoting tumor angiogenesis. In line with this hypothesis, the depletion of PCBP2 notably abolished the EGFR-driven tumor angiogenesis *in vivo* (Figure S10).

### PCBP2 depletion impedes OSCC angiogenesis by disrupting the secretion of miRNAs via sEVs

**Figure 6:**
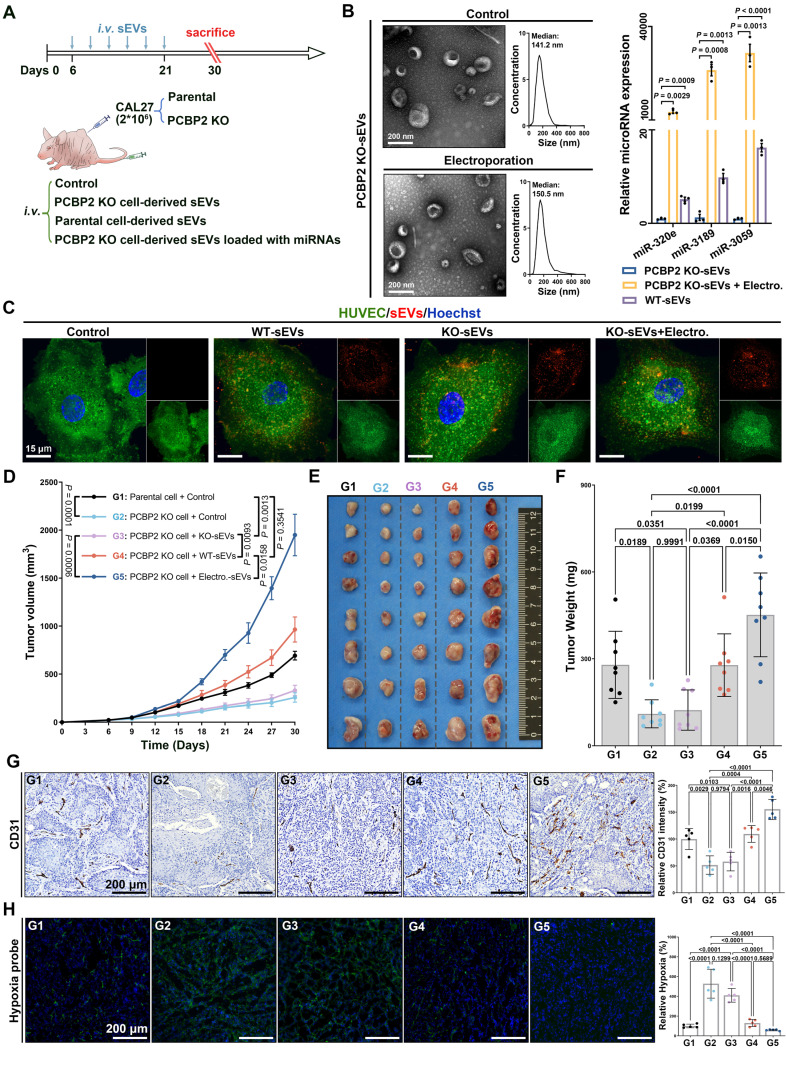
** PCBP2 depletion impedes OSCC angiogenesis by disrupting the secretion of miRNAs via sEVs. (A)** Experimental schema for assessing the role of PCBP2-mediated miRNA secretion via sEVs in tumor angiogenesis with a murine OSCC model. **(B)** Representative TEM and NTA results of sEVs with or without miRNA loading through electroporation (left panel), and comparing the content of miR-320e, miR-3189 and miR-3059 in the indicated sEV types by RT-qPCR (right panel). **(C)** The representative images of Clathrin-EGFP-HUVECs incubated with dye control (Control), parental CAL27 cells-derived sEVs (WT-sEVs), PCBP2-depleted CAL27 cells-derived sEVs (KO-sEVs), PCBP2-depleted CAL27 cells-derived sEVs which were reloaded with miRNA mimics through electroporation (KO-sEVs+Electro.), all sEVs were prelabeled with CellMask DeepRed. Scale bar, 15 μm. **(D)** The tumor growth of CAL27 xenograft tumors in BALB/c nude mice with indicated treatment, sEVs from PCBP2-depleted CAL27 cells (KO-sEVs), sEVs from parental CAL27 cells (WT-sEVs), sEVs from PCBP2-depleted CAL27 cells were loaded with miRNA mimics through electroporation (Electro.-sEVs). The CAL27 xenograft tumors were harvested **(E)** and weighed **(F)** at the indicated timepoint. **(G)** The representative images of CD31^+^ vessels in the tumor tissues. Scale bar, 200 μm. **(H)** The representative images of Hypoxia probe stain in the tumor tissues. Scale bar, 200 μm.

In the end, we verified the significance of PCBP2-driven miRNA secretion via sEVs in promoting tumor angiogenesis *in vivo*. Mouse OSCC models were established by injecting parental or PCBP2-knockout CAL27 cells subcutaneously into nude mice, followed by administration of sEVs via the tail vein (Figure 6A). We employed electroporation to encapsulate three typical GGGU motif-containing miRNAs into sEVs derived from PCBP2-depleted CAL27 cells. Consistent with numerous prior studies, this strategy effectively elevated the abundance of miR-320e, miRNA-3189 and miR-3059 within sEVs, while preserving the sEVs' intrinsic properties (Figure 6B). Notably, neither PCBP2 knockout nor electroporation strategy affected the uptake of sEVs by ECs (Figure 6C), just as we previously reported. Knocking out PCBP2 in CAL27 cells did not alter their proliferation and apoptosis *in vitro* (Figure S11), but notably inhibited the tumor growth *in vivo* (Figure 6D-F). Compared to sEVs derived from PCBP2-depleted CAL27 cells (KO-sEVs), reinfusion of CAL27 cells-derived sEVs (WT-sEVs) effectively reversed the growth disadvantage of PCBP2 knockout tumors. Importantly, augmenting the levels of target miRNAs (miR-320e, miRNA-3189 and miR-3059) within KO-sEVs (Electro.-sEVs) notably enhanced the growth of PCBP2 knockout tumors (Figure 6D-F). Deletion of PCBP2 markedly decreased intra-tumoral microvessel density, resulting in more focal necrosis within the tumor tissues (Figure 6G). WT-sEVs, but not KO-sEVs, effectively restored microvessel density within the tumor. However, reloading the target miRNAs into KO-sEVs more significantly promoted tumor angiogenesis (Figure 6G). Consistent with this, more severe hypoxia was observed in tumors with PCBP2 depletion, and WT-sEVs significantly improved intratumor hypoxia, while the effects of Electro-sEVs was more dramatic (Figure 6H). Altogether, these results suggest that PCBP2 depletion impedes OSCC angiogenesis mainly by disrupting the secretion of specific miRNAs via sEVs.

## Discussion

EGFR, a member of the HER/ErbB family of receptor tyrosine kinases, is involved in essential cellular processes such as cell proliferation, survival, and differentiation 1. Overexpression of EGFR confers a malignant phenotype, characterized by rapid population expansion and mass increasement 38. This leads to heightened requirements for both nutrient supply and outward expansion. Thus, pathological angiogenesis is initiated early in the development of solid tumors 13, 39. The results of numerous foundational studies and clinical trials have consistently highlighted the significant association between EGFR signaling and tumor angiogenesis 12, 39-41. These investigations have underscored the pivotal roles played by key proteins, including pro-angiogenic growth factors (VEGF, PDGF, and IL-8), matrix Metalloproteinases and EGFR itself. Recent studies have revealed that EV-mediated delivery of biomolecules can play a more stable, efficient and broad role 18, 24. This property may be even more important for nucleic acids, which were secreted into the extracellular environment primarily after being encapsulated within EVs 18. For the first time, we uncovered how EGFR overexpression governs miRNA contents within EVs derived from tumor cells, specifically focusing on their functional roles and the sorting mechanisms involved. This study provides novel evidence highlighting EGFR's role in shaping the tumor microenvironment through precise regulatory mechanisms involving miRNA secretion.

The biogenesis of miRNA involves a complex and highly coordinated process that ensures precise control over miRNA level and activity in cells 42. Pri-miRNA transcribed by RNA polymerase is processed by the microprocessor complex (Drosha and DGCR8) to form pre-miRNA, which is exported to the cytoplasm by Exportin-5 and cleaved by Dicer into a miRNA duplex. The mature miRNA guides RNA-induced silencing complex (RISC) to target mRNAs through sequence complementarity, leading to mRNA degradation or translational repression. Shen et al. reported that EGFR could modulate miRNA maturation in response to hypoxia through phosphorylation of argonaute 2 (AGO2) in multiple cancer cell lines 34. Our data also displayed the substantial impacts of increased EGFR level on the type and abundance of miRNAs in OSCC cells 43. In addition to activating specific sorting mechanisms, the regulation of miRNA biogenesis laid another foundation for the secretion of pathogenic miRNAs driven by EGFR. miRNAs offer several advantages over proteins, including smaller size, more efficient production, better conservation, and a broader range of targets. These strengths likely contribute to the thriving interests of miRNAs in pathological mechanisms, diagnostic strategy, and therapeutic innovation 42, 44.

Recently, the discovery of new regulatory mechanisms enabling cells to selectively control their miRNA secretion has significantly advanced research on EVs 31-33. The differences in relative abundance of miRNAs between EVs and the original cells have been argued as evidence towards specific, active mechanisms by which the miRNA is packaged into EVs. Similar to the loading of protein cargos, the sorting of miRNAs into EVs also depends on the cooperation of substrate and regulator. Specific characteristics of miRNAs, such as sequence motifs and modifications, which also impact RNA biogenesis and stability, are believed to favor their secretion via EVs under certain conditions 32. Sorting proteins can generally be categorized into RNA-binding proteins (RBPs) and non-RBPs. Those involved in the miRNA biogenesis also belong to the RBPs category, such as AGO2. Tan et al. demonstrated that KRAS overexpression promotes sorting of AGO2-associated miRNAs into EVs 45. Other reported RBPs include HnRNBP21, HuR, YBX1, and so on. PCBP2, the sorting regulator identified in this study, is also an RBP. Non-classical RBPs such as Alix, lupus La protein, and Arc1 have also been revealed to affect the secretion of specific miRNAs via EVs. These proteins may recognize miRNAs through non-direct binding or unreported RNA binding capabilities 18. Various regulators exhibit varying binding preferences for different miRNAs, although a comprehensive understanding of these specific binding interactions has yet to be established.

PCBP2, a member of the poly(C)-binding protein (PCBP) family, plays a crucial role in posttranscriptional and translational regulation by binding to single-stranded poly(C) sequences in target mRNAs 46, 47. Many studies have shown that PCBP2 expression is elevated in various cancers. It is considered as an oncogene that contributes to tumorigenesis, cancer cell development, and metastasis. PCBP2 stabilizes the mRNA of the ferroptosis inhibitor SLC7A11, which inhibits ferroptosis in tumors and accelerates bladder cancer progression 48. Research by Han et al. revealed that PCBP2 increases FHL3 expression by stabilizing its mRNA, thereby promoting glioma initiation and progression 47. Our study broadens the understanding of PCBP2's RNA regulatory functions by exploring its role in miRNA secretion, offering new insights into its involvement in tumor progression.

In our study, given that PCBP2 is not a recognized membrane protein, another remaining question following PCBP2's recognition of GGGU motif-containing miRNAs is how the miRNA-PCBP2 complex is incorporated into the interior of vesicles. While this process has not been systematically investigated, previous studies have provided some insights. Overexpression of Caveolin-1, a membrane protein which regulates receptor-independent endocytosis by organizing lipid rafts, increased the levels of hnRNPA2B1-associated miRNAs in EVs isolated from hyperoxia-treated cells 31, 32. Inhibition of nSMase2, another component that regulates cell membrane invagination, significantly reduced the levels of miR-16/146a within exosomes, while having no effect on their levels in the original cells 49. In addition, some studies have also revealed the participation of ESCRT-machinery in miRNA secretion. Enhancing the expression of vacuolar protein sorting-associated protein 4 (Vps4A) in hepatocellular carcinoma cells (HCC) led to increased exosomal levels of miR-27b-3p and miR-92a-3p 50. Conversely, inhibiting Vps4A in HEK293 cells resulted in decreased levels of EV-carried miR-92a and miR-150 51. Alix promotes AGO2-mediated miRNA sorting by direct binding in human liver stem-like cells 52. In our results of miRNA pull-down, we also identified several proteins associated with the regulation of EV biogenesis, including members of the ESCRT complex, small GTPases, cytoskeletal proteins, and other membrane components (Clathrin, Caveolin-1, Annexin A1). Further investigations into these candidates will aid in understanding how PCBP2-mediated miRNA sorting is spatiotemporally coupled with EV biogenesis.

The widespread employment of RNA sequencing has revealed that EVs or EV-like particles can harbor a diverse array of RNA biotypes, including mRNA, miRNA, lncRNA, Y-RNA, tRNA, and rRNA, either in full-length form or as fragments 53. Despite the identification of these RNA subtypes across different studies, discrepancies in conclusion often arise upon closer examination. These conflicts possibly stem from technical variations among studies, including differences in the cell types, the purification methods and the specific EV sub-populations under investigation. The identification of these diverse RNA types within EVs unwittingly echoes the initial perception of EVs as vehicles of cellular waste disposal. In fact, this indicates the speculation about whether the RNA cargos carried by EVs retain functional activity at all. Achieving clarity on these aspects is essential for elucidating the mechanisms underlying EV function and their implications in intercellular communication. Our study demonstrated that disrupting angiogenesis-associated miRNA sorting through PCBP2 depletion notably reduced the microvessel density in murine tumor tissues. Conversely, transfusion of EVs with reloaded target miRNAs effectively reversed this inhibition both *in vitro* and *in vivo*. More importantly, based on the established single particle tracking (SPT) platform, we previously recorded the whole dynamic process of OSCC-EVs delivering pro-angiogenic miRNA to HUVECs in real time and *in situ* at the level of single vesicle 43. Those findings provided direct evidence for EV-miRNA-mediated pro-angiogenesis. Since RNA sequencing experiments typically analyze bulk preparations of EVs, they provide limited insight into the heterogeneity of RNA distribution among individual EVs. The previously established SPT platform is also promising for dissecting the sorting process at the single-EV level.

In summary, PCBP2 is the first identified sorting protein responsible for the EGFR-driven miRNA secretion via EVs. This finding provides novel insight into how EGFR overexpression governs the tumor angiogenesis in various epithelial malignancies. Although certain details, such as the transcriptional activation of PCBP2 by EGFR and the validation of the PCBP2-miRNA complex in OSCC patients, still lack direct evidence, this study represents a significant contribution to the ongoing topic of EGFR-driven tumor angiogenesis. Furthermore, the EGFR-induced PCBP2-mediated sorting of miRNAs holds promise for advancing diagnostic and therapeutic strategies in various pathological conditions.

## Supplementary Material

Supplementary figures and table.

## Figures and Tables

**Table 1:** Primer sequences for RT-qPCR

For mRNA			
**Gene**	**Forward**		**Reverse**
*β-actin*	CATGTACGTTGCTATCCAGGC		CTCCTTAATGTCACGCACGAT
*PCBP2*	GCGCAGATCAAAATTGCGAAC		ATATTGAGCCAGGCTAATGCTG
pre*PCBP2*#1	TAGGCTCTGAAATTGCTGCTGG		GCCAGCAATAGTGATGGCC
pre*PCBP2*#2	GATTCTTAGACCTAGCCATGCAACTCT		AATTTTGATCTGCGCCCCAGAC
**For miRNA**			
**microRNA**		**Primer**	
has-miR-1268b		GTGGTGGTGGGGGTGAAA	
has-miR-1303		TTTAGAGACGGGGTCTTGCTCTAA	
has-miR-1909-3p		GGGCCGGGTGCTCACCGAAA	
has-miR-3059-5p		TTTCCTCTCTGCCCCATAGGGTGTAA	
has-miR-320e		AAAGCTGGGTTGAGAAGGAAA	
has-miR-3189-3p		TGGGTCTGATGGGGTAGAAAA	
has-miR-3195		CCGGGCCCGGGTTAA	
has-miR-5003-5p		CTTTTCTAGGTTGTTGGGGAAA	
has-miR-6129		TGAGGGAGTTGGGTGTATAA	
has-miR-6835-5p		GGGTAGAAAGTGGCTGAAGAAAA	
			

## References

[1] Selvaggi G, Novello S, Torri V, Leonardo E, De Giuli P, Borasio P (2004). Epidermal growth factor receptor overexpression correlates with a poor prognosis in completely resected non-small-cell lung cancer. Ann Oncol.

[2] Spindler KL, Lindebjerg J, Nielsen JN, Olsen DA, Bisgård C, Brandslund I (2006). Epidermal growth factor receptor analyses in colorectal cancer: a comparison of methods. Int J Oncol.

[3] Watanabe K, Tachibana O, Sata K, Yonekawa Y, Kleihues P, Ohgaki H (1996). Overexpression of the EGF receptor and p53 mutations are mutually exclusive in the evolution of primary and secondary glioblastomas. Brain Pathol.

[4] Grandis JR, Tweardy DJ (1993). Elevated levels of transforming growth factor alpha and epidermal growth factor receptor messenger RNA are early markers of carcinogenesis in head and neck cancer. Cancer Res.

[5] Bell DW, Lynch TJ, Haserlat SM, Harris PL, Okimoto RA, Brannigan BW (2005). Epidermal growth factor receptor mutations and gene amplification in non-small-cell lung cancer: molecular analysis of the IDEAL/INTACT gefitinib trials. J Clin Oncol.

[6] Clarke TL, Tang R, Chakraborty D, Van Rechem C, Ji F, Mishra S (2020). Histone lysine methylation dynamics control EGFR DNA copy-number amplification. Cancer Discov.

[7] Fan QW, Cheng CK, Gustafson WC, Charron E, Zipper P, Wong RA (2013). EGFR phosphorylates tumor-derived EGFRvIII driving STAT3/5 and progression in glioblastoma. Cancer Cell.

[8] Zhang S, Jia X, Dai H, Zhu X, Song W, Bian S (2024). SERPINE2 promotes liver cancer metastasis by inhibiting c-Cbl-mediated EGFR ubiquitination and degradation. Cancer Commun (Lond).

[9] Kokai Y, Myers JN, Wada T, Brown VI, LeVea CM, Davis JG (1989). Synergistic interaction of p185c-neu and the EGF receptor leads to transformation of rodent fibroblasts. Cell.

[10] Cho BC, Lu S, Felip E, Spira AI, Girard N, Lee JS (2024). Amivantamab plus Lazertinib in previously untreated EGFR-mutated advanced NSCLC. N Engl J Med.

[11] Wang Y, Liu C, Chen H, Jiao X, Wang Y, Cao Y (2024). Clinical efficacy and identification of factors confer resistance to afatinib (tyrosine kinase inhibitor) in EGFR-overexpressing esophageal squamous cell carcinoma. Signal Transduct Target Ther.

[12] Nilsson MB, Robichaux J, Herynk MH, Cascone T, Le X, Elamin Y (2021). Altered regulation of HIF-1α in naive- and drug-resistant EGFR-mutant NSCLC: implications for a vascular endothelial growth factor-dependent phenotype. J Thorac Oncol.

[13] Ronca R, Benkheil M, Mitola S, Struyf S, Liekens S (2017). Tumor angiogenesis revisited: Regulators and clinical implications. Med Res Rev.

[14] Han M, Sun H, Zhou Q, Liu J, Hu J, Yuan W (2023). Effects of RNA methylation on Tumor angiogenesis and cancer progression. Mol Cancer.

[15] Ciardiello F, Troiani T, Bianco R, Orditura M, Morgillo F, Martinelli E (2006). Interaction between the epidermal growth factor receptor (EGFR) and the vascular endothelial growth factor (VEGF) pathways: a rational approach for multi-target anticancer therapy. Ann Oncol.

[16] Wang Y, Wang L, Chen C, Chu X (2018). New insights into the regulatory role of microRNA in tumor angiogenesis and clinical implications. Mol Cancer.

[17] Fang JH, Zhou HC, Zeng C, Yang J, Liu Y, Huang X (2011). MicroRNA-29b suppresses tumor angiogenesis, invasion, and metastasis by regulating matrix metalloproteinase 2 expression. Hepatology.

[18] O'Brien K, Breyne K, Ughetto S, Laurent LC, Breakefield XO (2020). RNA delivery by extracellular vesicles in mammalian cells and its applications. Nat Rev Mol Cell Biol.

[19] Wong SWK, Tey SK, Mao X, Fung HL, Xiao ZJ, Wong DKH (2023). Small extracellular vesicle-derived vWF induces a positive feedback loop between tumor and endothelial cells to promote angiogenesis and metastasis in hepatocellular carcinoma. Adv Sci (Weinh).

[20] Sandim V, Monteiro RQ (2020). Extracellular vesicle fingerprinting: the next generation for cancer diagnosis?. Signal Transduct Target Ther.

[21] Debnath K, Heras KL, Rivera A, Lenzini S, Shin JW (2023). Extracellular vesicle-matrix interactions. Nat Rev Mater.

[22] Melo SA, Luecke LB, Kahlert C, Fernandez AF, Gammon ST, Kaye J (2015). Glypican-1 identifies cancer exosomes and detects early pancreatic cancer. Nature.

[23] Chen G, Huang AC, Zhang W, Zhang G, Wu M, Xu W (2018). Exosomal PD-L1 contributes to immunosuppression and is associated with anti-PD-1 response. Nature.

[24] Kalluri R, LeBleu VS (2020). The biology, function, and biomedical applications of exosomes. Science.

[25] Han T, Chen L, Li K, Hu Q, Zhang Y, You X (2023). Significant CircRNAs in liver cancer stem cell exosomes: mediator of malignant propagation in liver cancer?. Mol Cancer.

[26] Vietri M, Radulovic M, Stenmark H (2020). The many functions of ESCRTs. Nat Rev Mol Cell Biol.

[27] Guan L, Wu B, Li T, Beer LA, Sharma G, Li M (2022). HRS phosphorylation drives immunosuppressive exosome secretion and restricts CD8(+) T-cell infiltration into tumors. Nat Commun.

[28] Wei D, Zhan W, Gao Y, Huang L, Gong R, Wang W (2021). RAB31 marks and controls an ESCRT-independent exosome pathway. Cell Res.

[29] Preethi KA, Selvakumar SC, Ross K, Jayaraman S, Tusubira D, Sekar D (2022). Liquid biopsy: Exosomal microRNAs as novel diagnostic and prognostic biomarkers in cancer. Mol Cancer.

[30] Liu T, Zhang Q, Zhang J, Li C, Miao YR, Lei Q (2019). EVmiRNA: a database of miRNA profiling in extracellular vesicles. Nucleic Acids Res.

[31] Lee H, Li C, Zhang Y, Zhang D, Otterbein LE, Jin Y (2019). Caveolin-1 selectively regulates microRNA sorting into microvesicles after noxious stimuli. J Exp Med.

[32] Villarroya-Beltri C, Gutiérrez-Vázquez C, Sánchez-Cabo F, Pérez-Hernández D, Vázquez J, Martin-Cofreces N (2013). Sumoylated hnRNPA2B1 controls the sorting of miRNAs into exosomes through binding to specific motifs. Nat Commun.

[33] Shurtleff MJ, Temoche-Diaz MM, Karfilis KV, Ri S, Schekman R (2016). Y-box protein 1 is required to sort microRNAs into exosomes in cells and in a cell-free reaction. Elife.

[34] Shen J, Xia W, Khotskaya YB, Huo L, Nakanishi K, Lim SO (2013). EGFR modulates microRNA maturation in response to hypoxia through phosphorylation of AGO2. Nature.

[35] Nishida N, Mimori K, Mori M, Calin GA (2013). EGFR gets in the way of microRNA biogenesis. Cell Res.

[36] Ren JG, Zhang W, Liu B, Man QW, Xiong XP, Li C (2016). Clinical significance and roles in angiogenesis of circulating microparticles in oral cancer. J Dent Res.

[37] Love SL, Emerson JD, Koide K, Hoskins AA (2023). Pre-mRNA splicing-associated diseases and therapies. RNA Biol.

[38] Lee JK, Lee J, Kim S, Kim S, Youk J, Park S (2017). Clonal history and genetic predictors of transformation into small-cell carcinomas from lung adenocarcinomas. J Clin Oncol.

[39] Zhou Q, Xu CR, Cheng Y, Liu YP, Chen GY, Cui JW (2021). Bevacizumab plus erlotinib in Chinese patients with untreated, EGFR-mutated, advanced NSCLC (ARTEMIS-CTONG1509): A multicenter phase 3 study. Cancer Cell.

[40] Eskilsson E, Rosland GV, Talasila KM, Knappskog S, Keunen O, Sottoriva A (2016). EGFRvIII mutations can emerge as late and heterogenous events in glioblastoma development and promote angiogenesis through Src activation. Neuro Oncol.

[41] Koizumi H, Yamada T, Takeuchi S, Nakagawa T, Kita K, Nakamura T (2012). Hsp90 inhibition overcomes HGF-triggering resistance to EGFR-TKIs in EGFR-mutant lung cancer by decreasing client protein expression and angiogenesis. J Thorac Oncol.

[42] Shang R, Lee S, Senavirathne G, Lai EC (2023). microRNAs in action: biogenesis, function and regulation. Nat Rev Genet.

[43] Xia HF, Yu ZL, Zhang LJ, Liu SL, Zhao Y, Huang J (2023). Real-time dissection of the transportation and miRNA-release dynamics of small extracellular vesicles. Adv Sci (Weinh).

[44] Di Leva G, Garofalo M, Croce CM (2014). MicroRNAs in cancer. Annu Rev Pathol.

[45] McKenzie AJ, Hoshino D, Hong NH, Cha DJ, Franklin JL, Coffey RJ (2016). KRAS-MEK signaling controls Ago2 sorting into exosomes. Cell Rep.

[46] Yuan C, Chen M, Cai X (2021). Advances in poly(rC)-binding protein 2: Structure, molecular function, and roles in cancer. Biomed Pharmacother.

[47] Han W, Xin Z, Zhao Z, Bao W, Lin X, Yin B (2013). RNA-binding protein PCBP2 modulates glioma growth by regulating FHL3. J Clin Invest.

[48] Shen L, Zhang J, Zheng Z, Yang F, Liu S, Wu Y (2022). PHGDH inhibits ferroptosis and promotes malignant progression by upregulating SLC7A11 in bladder cancer. Int J Biol Sci.

[49] Kosaka N, Iguchi H, Yoshioka Y, Takeshita F, Matsuki Y, Ochiya T (2010). Secretory mechanisms and intercellular transfer of microRNAs in living cells. J Biol Chem.

[50] Wei JX, Lv LH, Wan YL, Cao Y, Li GL, Lin HM (2015). Vps4A functions as a tumor suppressor by regulating the secretion and uptake of exosomal microRNAs in human hepatoma cells. Hepatology.

[51] Jackson CE, Scruggs BS, Schaffer JE, Hanson PI (2017). Effects of inhibiting VPS4 support a general role for ESCRTs in extracellular vesicle biogenesis. Biophys J.

[52] Iavello A, Frech VS, Gai C, Deregibus MC, Quesenberry PJ, Camussi G (2016). Role of Alix in miRNA packaging during extracellular vesicle biogenesis. Int J Mol Med.

[53] Dellar ER, Hill C, Melling GE, Carter DRF, Baena-Lopez LA (2022). Unpacking extracellular vesicles: RNA cargo loading and function. J Extracell Biol.

