# Supplementary material for: PCBP2-dependent secretion of miRNAs via extracellular vesicles contributes to the EGFR-driven angiogenesis
Source: Theranostics. 2025 Jan 1;15(4):1255–71. doi: 10.7150/thno.102391 (PMC11729547; doi:10.7150/thno.102391)
Supplement: Supplementary file 1 — Supplementary figures and table. [file thnov15p1255s1.pdf]

Supporting Information

PCBP2-dependent secretion of miRNAs via extracellular vesicles contributes to the EGFR-driven angiogenesis

Hou-Fu Xia [1,2], Xiao-Le Wang[1], He-Jing Zhang[1], Kui-Ming Wang[1], Lin-Zhou Zhang[1], Yang Yang[1], Xin Shi[5*], Gang Chen[1,2,3,4*]

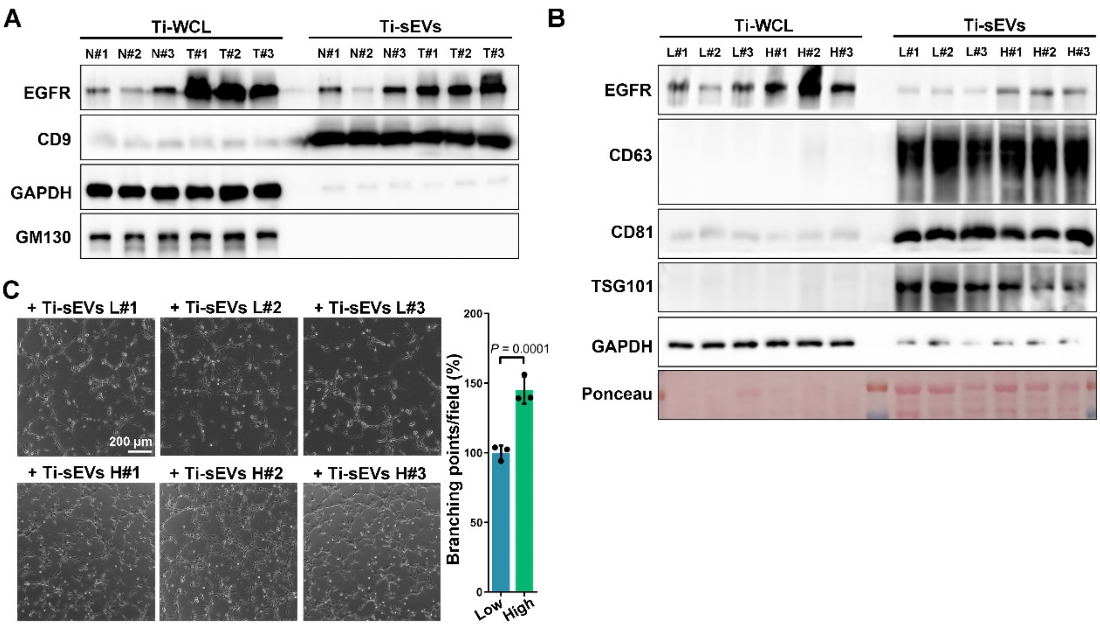

Figure S1. The pro-angiogenic activity of OSCC Ti-sEVs. (A) Characterizing the sEVs isolated from tumor tissues and normal mucosa of OSCC patients by western blotting, whole cell lysate (WCL). (B) Characterizing the sEVs isolated from tumor tissues of OSCC patients with different EGFR expression levels by western blotting, 3 OSCC patients with relatively low EGFR expression (L#1, L#2, L#3), 3 OSCC patients with relatively high EGFR expression (H#1, H#2, H#3), whole cell lysate (WCL). (C) Comparing the pro-angiogenic ability of Ti-sEVs isolated from tumor tissues of OSCC patients with different EGFR expression levels through tube formation assay, 3 OSCC patients with relatively low EGFR expression (L#1, L#2, L#3), 3 OSCC patients with relatively high EGFR expression (H#1, H#2, H#3).

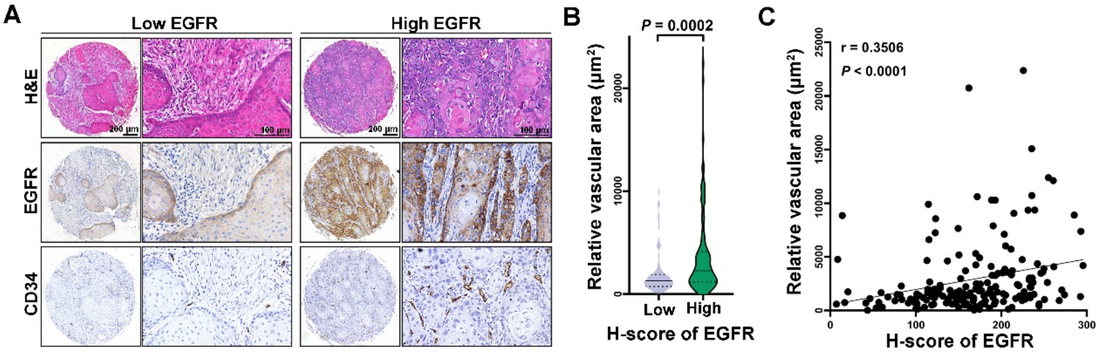

Figure S2. The correlation between EGFR level and microvessel density in OSCC. (A) Representative images of IHC stain of EGFR and CD34 in OSCC tissues. (B) Quantifying the microvessel density in OSCC patients with a low or high expression level of EGFR. (C) The positive correlation between EGFR level and microvessel density in OSCC, n = 175.

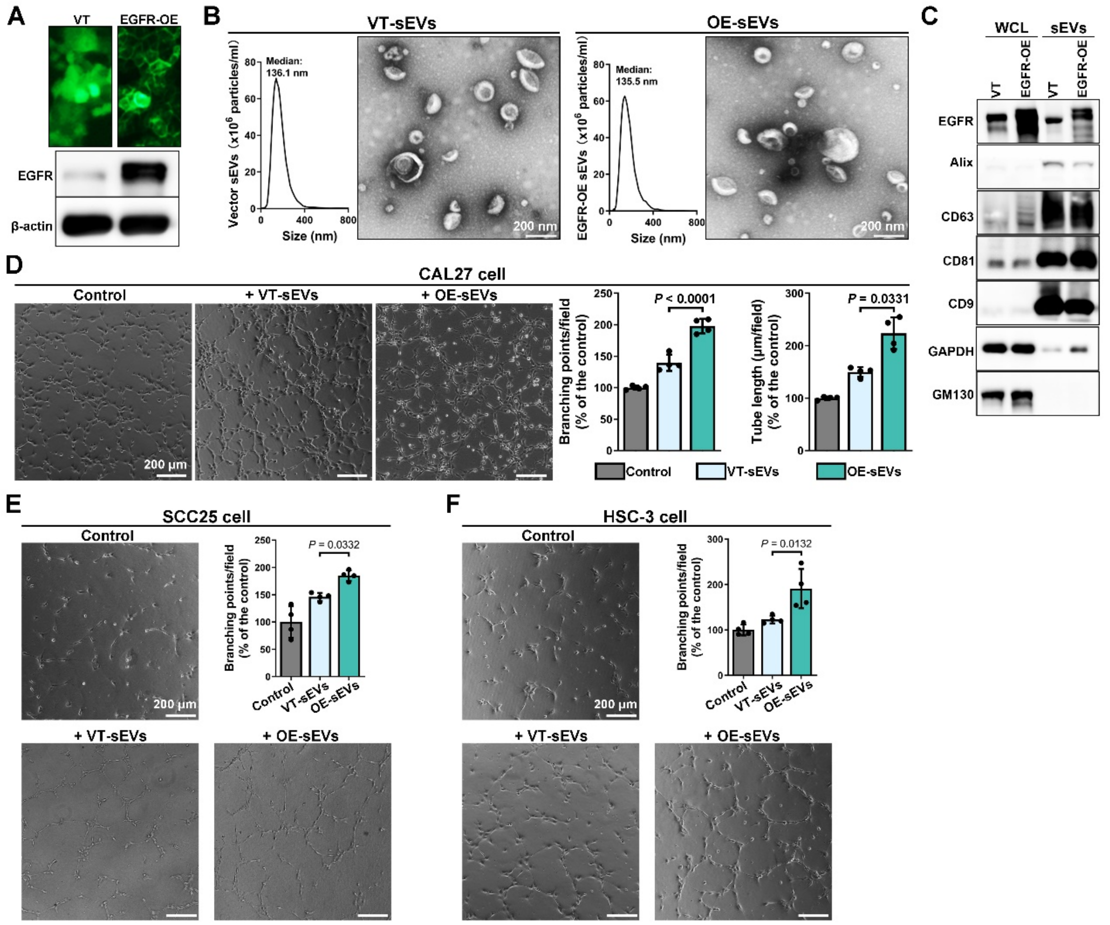

Figure S3. The pro-angiogenic activity of sEVs-derived from EGFR-overexpressed CAL27 cells. (A) Constructing EGFR-overexpressed CAL27 cell lines with lentivirus. (B) Characterizing the sEVs isolated from control (VT-sEVs) and EGFR-overexpressed CAL27 cells (OE-sEVs) with NTA and TEM. (C) Characterizing the sEVs isolated from control (VT) and EGFR-overexpressed CAL27 cells (EGFR-OE) with western blotting, whole cell lysate (WCL). (D) Comparing the pro-angiogenic ability of sEVs

isolated from control (VT-sEVs) and EGFR-overexpressed CAL27 cells (OE-sEVs) through tube formation assay. (E) Comparing the pro-angiogenic ability of sEVs isolated from control (VT-sEVs) and EGFR-overexpressed SCC25 cells (OE-sEVs) through tube formation assay. (F) Comparing the pro-angiogenic ability of sEVs isolated from control (VT-sEVs) and EGFR-overexpressed HSC-3 cells (OE-sEVs) through tube formation assay.

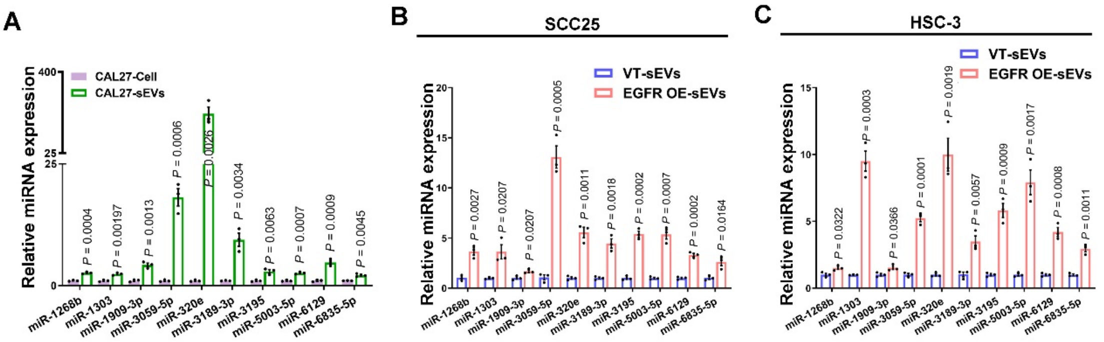

Figure S4. The enrichment of GGGU motif-containing miRNAs in sEVs derived from OSCC cells. (A) Comparing the levels of 10 GGGU motif-containing miRNAs in CAL27 cells (CAL27-Cell) and the derived sEVs (CAL27-sEVs) by RT-qPCR. (B) Comparing the levels of 10 GGGU motif-containing miRNAs in sEVs derived from SCC25 cells with (EGFR OE-sEVs) or without (VT-sEVs) EGFR-overexpression by RT-qPCR. (C) Comparing the levels of 10 GGGU motif-containing miRNAs in sEVs derived from HSC-3 cells with (EGFR OE-sEVs) or without (VT-sEVs) EGFR-overexpression by RT-qPCR.

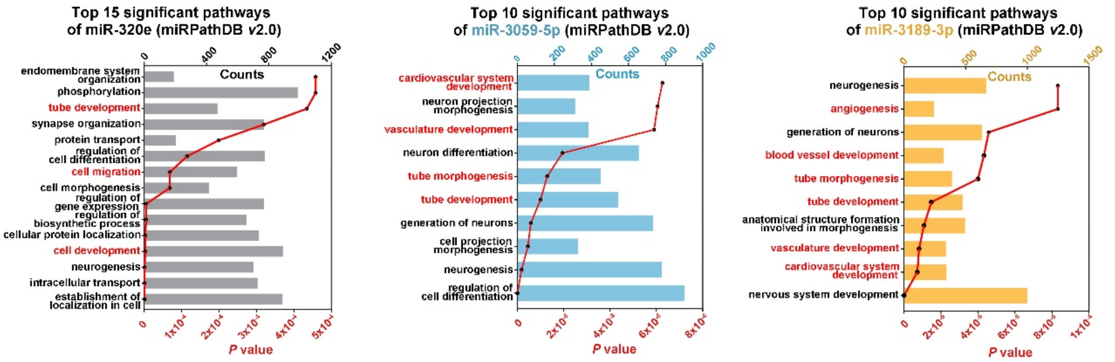

Figure S5. Analyzing the top target pathways of miR-320e, miR-3189-3p and miR-3059-5p involved respectively. The miRNA Pathway Dictionary Database (miRPathDB).

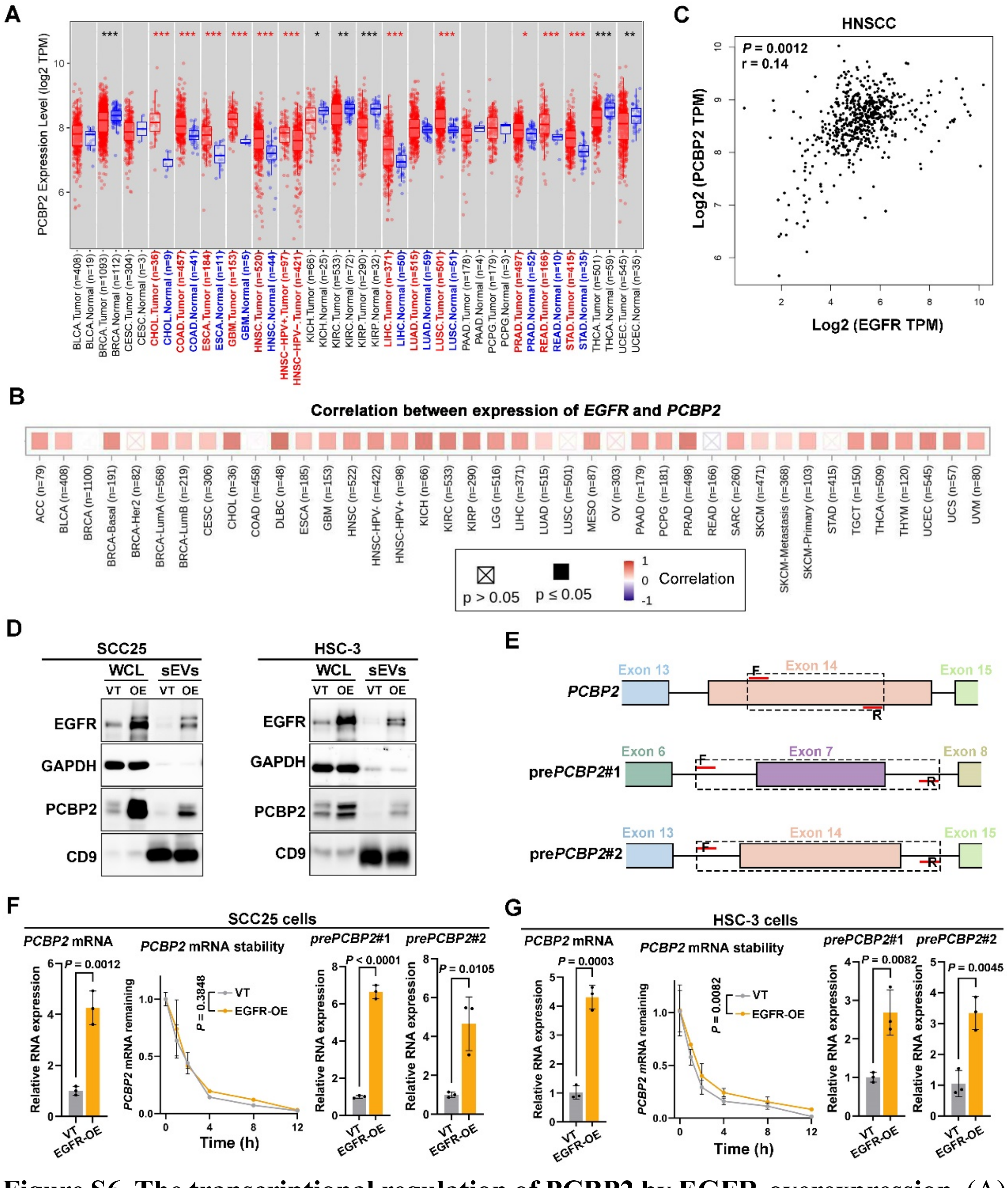

Figure S6. The transcriptional regulation of PCBP2 by EGFR-overexpression. (A) The mRNA level of *PCBP2* in different malignancies. (B) The positive correlation between mRNA levels of *EGFR* and *PCBP2* in different malignancies (TIMER2.0). (C) The correlation between mRNA levels of *EGFR* and *PCBP2* in HNSCC was analyzed in TCGA. (D) EGFR-overexpression up-regulates the PCBP2 protein levels in SCC25 and HSC-3 cells. (E) Schematic illustration of primers used to detect mature mRNA (*PCBP2*) and precursor mRNA (pre*PCBP2*#1/#2) of *PCBP2*, the black dashed box indicates the transcription products. (E) The mature mRNA level, mature mRNA stability and precursor mRNA content of *PCBP2* in SCC25 cells were detected by RT-PCR assay. (F) The mature mRNA level, mature mRNA stability and precursor mRNA content of *PCBP2* in HSC-3 cells were detected by RT-PCR assay.

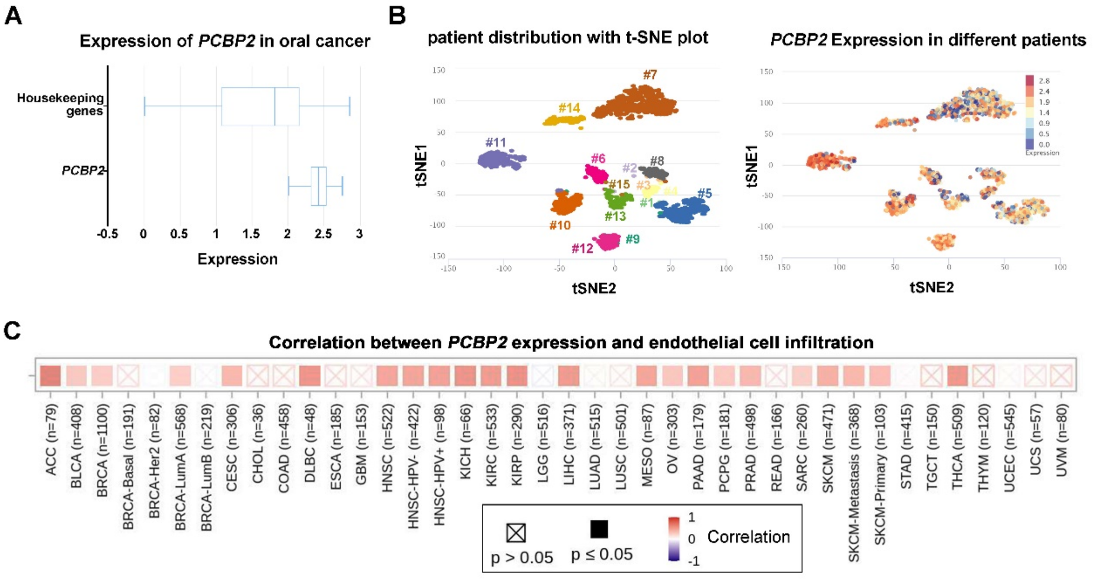

Figure S7. The association between *PCBP2* level and tumor angiogenesis was analyzed in public databases. (A) The level of *PCBP2* was significantly up-regulated in oral cancer patients, result obtained from the reported database (CancerSEA). (B) The consistent expression of *PCBP2* in tumorous cells of 15 oral cancer patients (CancerSEA). (C) The correlation between *PCBP2* levels and endothelial cell infiltration in different malignancies (TIMER 2.0).

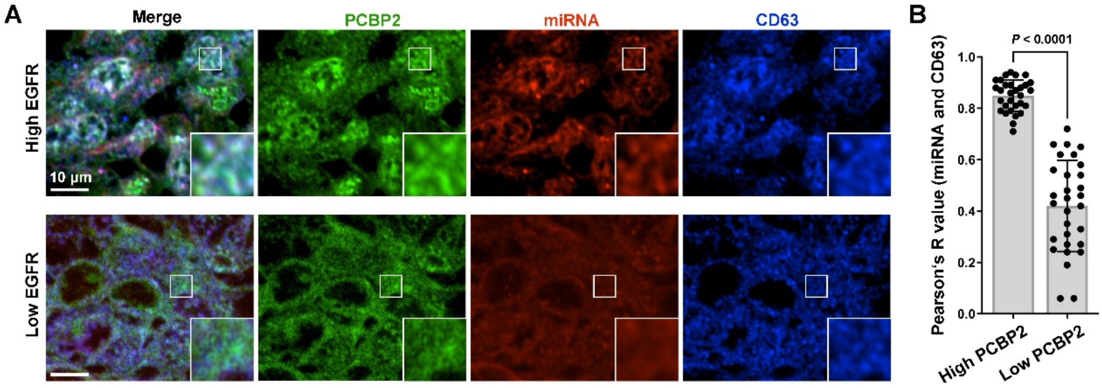

Figure S8. FISH experiments in clinical OSCC tissues demonstrate the regulatory effects of PCBP2 on miRNA loading. (A) Representative images of immunofluorescence staining of PCBP2, miR-3189-3p and CD63 in human OSCC tissues with different EGFR expression levels. Scale bar, 10 μm. (B) Statistical analysis about the co-localization efficiency (Pearson's value) of miR-3189-3p and CD63 in human OSCC tissues with different PCBP2 expression levels.

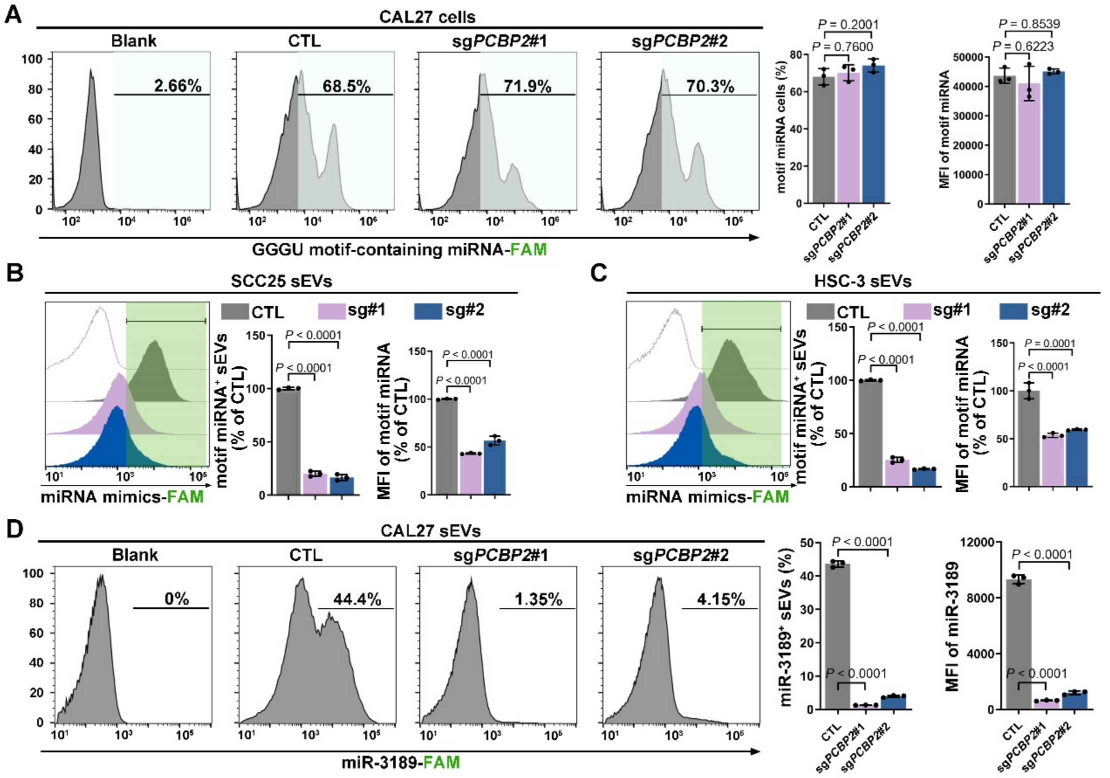

Figure S9. The effects of PCBP2 depletion on the secretion of miRNAs via sEVs. (A) Comparing the transfection efficiency of GGGU motif-containing miRNA mimics into CAL27 cells with or without PCBP2 depletion. Comparing the secretion of GGGU motif-containing miRNA mimics (miRNA mimics-FAM) via sEVs in SCC25 cells (B) and HSC-3 cells (C) with (sg#1/#2) or without (CTL) PCBP2 depletion by highly sensitive flow cytometry. (D) Comparing the abundance of FAM-labeled miR-3189 mimics in sEVs isolated from CAL27 cells with (sg*PCBP2*#1/#2) or without (CTL) PCBP2 depletion.

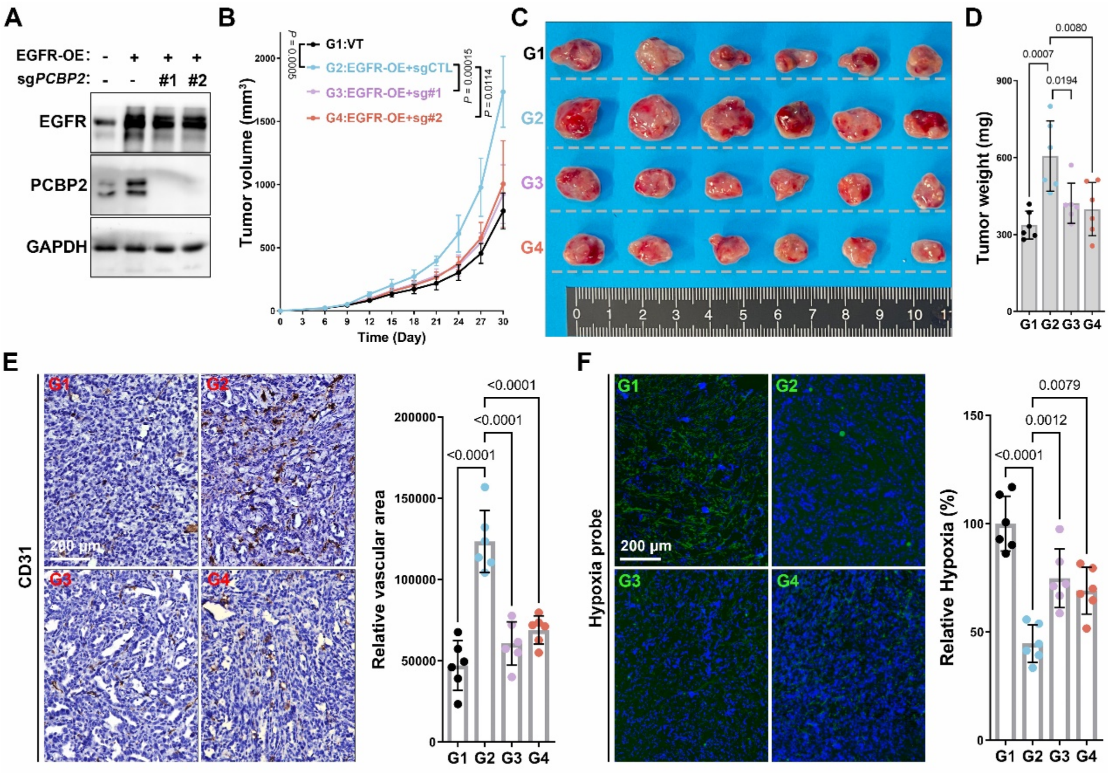

Figure S10. PCBP2-depletion impairs the EGFR-driven tumor angiogenesis. (A) Depleting PCBP2 in the EGFR-overexpressed CAL27 cell lines with CRISPR-Cas9 technology. (B) The tumor growth of different CAL27 xenograft tumors in BALB/c nude mice, Control CAL27 cells (G1: VT), EGFR-overexpressed CAL27 cell without PCBP2 depletion (G2: EGFR-OE+sgCTL), EGFR-overexpressed CAL27 cell with PCBP2 depletion (G3: EGFR-OE+sg#1, G4: EGFR-OE+sg#2). The CAL27 xenograft tumors were harvested (C) and weighed (D) at the indicated timepoint. (E) The representative images of CD31^{+} vessels in the tumor tissues. (F) The representative images of Hypoxia probe stain in the tumor tissues. Scale bar, 200 μm.

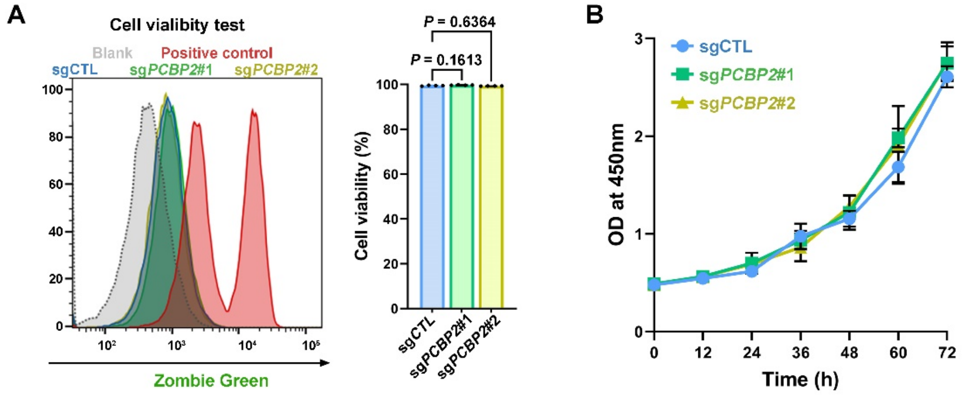

Figure S11. The effects of PCBP2 depletion on apoptosis (A) and proliferation (B) of CAL27 cells.

Table S1. The characteristics of OSCC patients

Number	Sex	Age	TNM
OSCC-1	Male	48	T1N0M0
OSCC-2	Female	77	T1N0M0
OSCC-3	Male	38	T1N0M0
OSCC-4	Male	29	T2N0M0
OSCC-5	Male	39	T2N0M0
OSCC-6	Male	77	T2N0M0
OSCC-7	Female	64	T2N0M0
OSCC-8	Male	59	T2N1M0
OSCC-9	Female	49	T2N0M0
OSCC-10	Male	63	T2N0M0
OSCC-11	Male	38	T1N0M0
OSCC-12	Male	51	T4N1M0
OSCC-13	Male	66	T4N2M0
OSCC-14	Male	58	T4N0M0
OSCC-15	Male	39	T4N0M0
OSCC-16	Male	64	T4N1M0
OSCC-17	Male	31	T2N1M0
OSCC-18	Male	52	T2N3M0
OSCC-19	Male	75	T2N0M0
OSCC-20	Female	70	T2N3M0
OSCC-21	Male	63	T3N0M0
OSCC-22	Male	62	T3N0M0
OSCC-23	Male	48	T4N2M0
OSCC-24	Male	31	T2N0M0
OSCC-25	Male	56	T3N0M0
OSCC-26	Male	48	T3N2M0
OSCC-27	Male	65	T3N3M0
OSCC-28	Male	82	T4N0M0
OSCC-29	Male	50	T4N0M0
OSCC-30	Female	55	T4N3M0
OSCC-31	Female	59	T2N0M0
OSCC-32	Male	53	T3N0M0
OSCC-33	Male	69	T3N0M0
OSCC-34	Male	62	T3N0M0
OSCC-35	Female	71	T2N0M0
OSCC-36	Male	68	T2N1M0
OSCC-37	Male	41	T3N1M0
OSCC-38	Male	68	T1N0M0

OSCC-39	Male	60	T2N0M0
OSCC-40	Female	60	T2N0M0
OSCC-41	Male	65	T2N0M0
OSCC-42	Male	63	T2N2M0
OSCC-43	Male	55	T2N0M0
OSCC-44	Male	61	T3N0M0
OSCC-45	Male	70	T3N3M0
OSCC-46	Female	64	T4N0M0
OSCC-47	Female	66	T4N2M0
OSCC-48	Male	48	T4N2M0
OSCC-49	Female	64	T2N0M0
OSCC-50	Male	58	T2N0M0
OSCC-51	Male	46	T2M0N0
OSCC-52	Male	46	T3N1MX
OSCC-53	Male	38	T2N0M0
OSCC-54	Male	55	T2N0M0
OSCC-55	Male	44	T1N0M0
OSCC-56	Female	44	T2N0M0
OSCC-57	Female	66	T1N0M0
OSCC-58	Female	57	T1N0M0
OSCC-59	Female	50	T2N0M0
OSCC-60	Male	46	T2N0M0
OSCC-61	Male	43	T2N0M1
OSCC-62	Male	70	T2N0M0
OSCC-63	Male	53	T2N0Mx
OSCC-64	Male	54	T4N0M0
OSCC-65	Male	57	T2N0M0
OSCC-66	Male	65	T2N1M0
OSCC-67	Female	58	T1N0M0
OSCC-68	Male	51	T3N0M0
OSCC-69	Male	46	TxN3M0
OSCC-70	Male	60	T3N2M0
OSCC-71	Female	69	T2N0M0
OSCC-72	Male	57	T2N1M0
OSCC-73	Male	60	T3N0M0
OSCC-74	Male	60	T2N0M0
OSCC-75	Female	37	T2N0M0
OSCC-76	Male	62	T3N2M0
OSCC-77	Male	47	T1N0M0
OSCC-78	Male	62	T2N0M0

OSCC-79	Male	46	T3N0M0
OSCC-80	Male	69	T2N1M0
OSCC-81	Male	49	T1N1M0
OSCC-82	Male	60	T4N1M0
OSCC-83	Female	55	T2N0M0
OSCC-84	Male	57	T2N0M0
OSCC-85	Male	73	T3N2M0
OSCC-86	Male	64	T2N1M0
OSCC-87	Male	56	T4aN2aM0
OSCC-88	Male	44	T2N2M0
OSCC-89	Male	58	T2N0M0
OSCC-90	Male	74	T3N2M0
OSCC-91	Male	48	T4aN1M0
OSCC-92	Male	55	T2N0M0
OSCC-93	Male	44	T2N0M0
OSCC-94	Male	50	T3N0M0
OSCC-95	Male	46	T2N0M0
OSCC-96	Male	43	T3N0M0
OSCC-97	Male	60	T2N0M0
OSCC-98	Male	41	T4aN2M0
OSCC-99	Male	59	T2N1M0
OSCC-100	Male	60	T2N0M0
OSCC-101	Female	64	T1N0M0
OSCC-102	Male	40	T2N0M0
OSCC-103	Male	53	T2N0M0
OSCC-104	Male	48	T2N2aM0
OSCC-105	Male	63	T1N0M0
OSCC-106	Female	43	T2N0M0
OSCC-107	Male	53	T2N0M0
OSCC-108	Male	60	T3N1M0
OSCC-109	Male	58	T4N2M0
OSCC-110	Male	65	T2N0M0
OSCC-111	Male	66	T1N0M0
OSCC-112	Male	53	T1M0N0
OSCC-113	Female	56	T4aN1Mx
OSCC-114	Male	50	T1N1M0
OSCC-115	Male	41	T1M0N0
OSCC-116	Male	44	T3N0M0
OSCC-117	Male	45	T1N0M0
OSCC-118	Male	59	T1N0M0

OSCC-119	Male	47	T2N0M0
OSCC-120	Male	51	T2N0M0
OSCC-121	Male	47	T2N0M0
OSCC-122	Male	64	T3N0M0
OSCC-123	Male	60	T4N2bM0
OSCC-124	Male	56	T4N1M0
OSCC-125	Female	55	T2N0M0
OSCC-126	Male	77	T4N1M0
OSCC-127	Male	55	T3N1M0
OSCC-128	Male	62	T3N0M0
OSCC-129	Male	62	T3N1M0
OSCC-130	Male	57	T4N2cM0
OSCC-131	Male	60	T2N0M0
OSCC-132	Male	31	T2N1M0
OSCC-133	Male	63	T2N0M0
OSCC-134	Male	76	T2N2M0
OSCC-135	Female	70	T3N2M0
OSCC-136	Male	54	T4N2M0
OSCC-137	Male	56	T4N1M0
OSCC-138	Male	54	T4N0M0
OSCC-139	Male	46	T4N0M0
OSCC-140	Male	71	T3N0M0
OSCC-141	Male	67	T3N0M0
OSCC-142	Female	55	T2N2M0
OSCC-143	Male	60	T4N1M0
OSCC-144	Female	77	T3N0M0
OSCC-145	Male	54	T2N0M0
OSCC-146	Male	63	T2N0M0
OSCC-147	Male	74	T3N2CM0
OSCC-148	Female	77	T2N0M0
OSCC-149	Male	58	T4N0M0
OSCC-150	Male	31	T3N1M0
OSCC-151	Male	36	T2N1M0
OSCC-152	Male	54	T4N1M0
OSCC-153	Female	68	T2N0M0
OSCC-154	Male	38	T4N2M0
OSCC-155	Male	66	T3N1M0
OSCC-156	Male	41	T2N1M0
OSCC-157	Male	58	T3N0M0
OSCC-158	Male	53	T3N1M0

OSCC-159	Male	64	T3N0M0
OSCC-160	Male	69	T2N0M0
OSCC-161	Male	61	T2N0M0
OSCC-162	Male	75	T3N0M0
OSCC-163	Male	31	T3N0M0
OSCC-164	Male	65	T2N0M0
OSCC-165	Male	61	T2N2M0
OSCC-166	Male	63	T2N2bM0
OSCC-167	Male	64	T4N0M0
OSCC-168	Female	70	T4N1M0
OSCC-169	Male	51	T4N1M0
OSCC-170	Male	64	T2N0M0
OSCC-171	Male	38	T4N1M0
OSCC-172	Male	82	T4N1M0
OSCC-173	Female	66	T2N1M0
OSCC-174	Male	75	T3N1M0
OSCC-175	Male	39	T2N1M0
